# ATF4 Transcriptionally Activates SHH to Promote Proliferation, Invasion, and Migration of Gastric Cancer Cells

**DOI:** 10.3390/cancers15051429

**Published:** 2023-02-23

**Authors:** Yang Wang, Muhammad Ali, Qi Zhang, Qiannan Sun, Jun Ren, Wei Wang, Dong Tang, Daorong Wang

**Affiliations:** 1 Department of General Surgery, General Surgery Institute of Yangzhou, Northern Jiangsu People’s Hospital, Clinical Medical College, Yangzhou University, Yangzhou 225009, China; 2 Department of General Surgery, Northern Jiangsu People’s Hospital, Yangzhou 225001, China; 3 General Surgery Institute of Yangzhou, Yangzhou University, Yangzhou 225009, China; 4 Yangzhou Key Laboratory of Basic and Clinical Transformation of Digestive and Metabolic Diseases, Yangzhou 225001, China

**Keywords:** transcriptional factor, ATF4, SHH, gastric cancer

## Abstract

**Simple Summary:**

Gastric cancer (GC) is the world’s third greatest cause of cancer-related death. Since the underlying pathogenic mechanisms are still unclear and only a limited number of specialized drugs have been developed, treating GC patients in clinical practice remains challenging. We observed that ATF4 was markedly upregulated in gastric cancer (GC) using immunohistochemistry and Western blotting assays in 80 paraffin-embedded GC samples and 4 fresh samples and para-cancerous tissues. The mechanism of ATF4 as a transcription factor in gastric cancer remains unclear. ATF4 knockdown using lentiviral vectors strongly inhibited the proliferation and invasion of GC cells. ATF4 upregulation using lentiviral vectors promoted the proliferation and invasion of GC cells. We observed that transcription factor ATF4 is bound to the promoter region of SHH to activate the Sonic Hedgehog pathway. Mechanistically, rescue assays showed that ATF4 regulated gastric cancer cells’ proliferation and invasive ability through SHH.

**Abstract:**

Activating transcription factor 4 (ATF4) is a DNA-binding protein widely generated in mammals, which has two biological characteristics that bind the cAMP response element (CRE). The mechanism of ATF4 as a transcription factor in gastric cancer affecting the Hedgehog pathway remains unclear. Here, we observed that ATF4 was markedly upregulated in gastric cancer (GC) using immunohistochemistry and Western blotting assays in 80 paraffin-embedded GC samples and 4 fresh samples and para-cancerous tissues. ATF4 knockdown using lentiviral vectors strongly inhibited the proliferation and invasion of GC cells. ATF4 upregulation using lentiviral vectors promoted the proliferation and invasion of GC cells. We predicted that the transcription factor ATF4 is bound to the SHH promoter via the JASPA database. Transcription factor ATF4 is bound to the promoter region of SHH to activate the Sonic Hedgehog pathway. Mechanistically, rescue assays showed that ATF4 regulated gastric cancer cells’ proliferation and invasive ability through SHH. Similarly, ATF4 enhanced the tumor formation of GC cells in a xenograft model.

## 1. Introduction

Gastric cancer (GC) is the world’s third greatest cause of cancer-related death, despite the fact that its incidence and mortality have reduced drastically over the previous 50 years [1]. There are around 1.2 million newly diagnosed instances of gastric cancer worldwide, with China accounting for 40%. Only 20% of gastric cancers are discovered in their early stages, with the majority being advanced, and the total 5-year survival rate is less than 50% [2]. A comprehensive therapy approach utilizing effective molecular target medicines should be investigated to improve patient prognosis significantly. Since the underlying pathogenic mechanisms are still unclear and only a limited number of specialized drugs have been developed, treating GC patients in clinical practice remains challenging [3]. Due to the fact that the underlying pathogenic mechanisms are still unclear and that only a limited number of specialized drugs have been developed, treating GC patients in clinical practice remains very challenging [4].

The activating transcription factor family shares a conserved basic-region leucine zipper domain and was first discovered in E1A-mediated transcriptional activation in 1987 [5]. It has been reported that over 20 ATF/CREB members exist in mammals, and some of these members play important roles in cancer progression. The activating transcription factor 2 (ATF2) is a member of the family of bZIP transcription factors that regulate transcription, remodel chromatin, and respond to DNA damage [6]. As one of the ATF/CREB transcription factors, activating transcription factor 3 (ATF3) can respond to a variety of stress signals, including anoxia, carcinogens, and genetically modified foods [7]. ATF6 protects from DNA damage and cell death in colon cancer cells [8]. Activating transcription factor 4 (ATF4) is a DNA-binding protein that is widely produced in mammals. The transcription factor has two biological characteristics that bind the cAMP response element (CRE): during an integrated stress response (ISR), as a master transcription factor, and as a regulator of metabolic and redox processes in normal cellular conditions [4,9,10]. As a transcriptional activator, ATF4 is abnormally expressed in many types of tumors by promoting the expression of downstream molecules. For example, breast cancer, colorectal cancer, prostate cancer, and ATF4 are all involved in tumorigenesis [11,12,13,14]. There is some evidence suggesting that ATF4 regulates eral metabolites, including amino acids and glucose, which promote cancer development [15]. Furthermore, evidence suggests that long-term leucine deprivation might inhibit mTORC1 activity through ATF4-mediated REDD1 and Sestrin2 upregulation [16]. Stress is an inevitable part of the growth process for cancer cells. The need for protein synthesis is increased by hyperproliferation [17]. The unfolded protein response (UPR), which is triggered by ER stress; the cellular response to low oxygen levels; and the amino acid response (AAR), which is triggered by amino acid deficiency, all share ATF4 as a common critical downstream effector protein [18,19,20]. Consequently, this contributes to the growth of malignancies. ATF4 is a critical regulator of the transcription of important genes required for the management of the adaptive function. According to studies, endoplasmic reticulum stress or amino acid deficiency triggers the eIF2α/ATF4 pathway, which controls the transcription [21]. However, the precise biochemical mechanism underlying ATF4’s contribution to gastric cancer is poorly understood.

Here, we discovered that the genomic DNA of ATF4 was commonly amplified in GC by evaluating GEPIA, whose RNA sequencing expression data of cancers and normal samples are from TCGA public databases (http://gepia.cancer-pku.cn/ (accessed on 15 May 2022)). Then, after analyzing the JASPA transcriptome database, we discovered that the SHH promoter region contains a large number of binding sites for the transcription factor ATF4.

The Hedgehog (Hh) gene family is known to regulate the development of stem cells. In addition, activation is responsible for the induction of GLI1 proto-oncogene and subsequent cellular proliferation. Sonic Hedgehog (SHH), one of the Hh family members, promotes carcinogenesis in the airway, and pancreatic epithelia are expressed in colonic stem cells. Some research demonstrated increased expression of SHH mRNA in human colonic adenocarcinomas and in a colorectal cell line with downstream increased expression of GLI1 mRNA, known to promote cell proliferation [22]. The deregulation of SHH signaling is often observed during tumor formation and progression and is detrimental to the cancerous process. The deregulation of the Hh signaling pathway is associated with developmental anomalies and cancer, including Gorlin syndrome, and sporadic cancers, such as basal cell carcinoma, medulloblastoma, pancreatic, breast, colon, ovarian, and small-cell lung carcinomas. The aberrant activation of the Hh signaling pathway is caused by mutations in the related genes or by the excessive expression of the Hh signaling molecules [23]. Significantly, SHH controls the development of vertebrate organs, such as the architecture of the brain and the creation of fingers on limbs. SHH continues to play a significant role in adult cells. It also regulates adult stem cells’ ability to divide, and several types of cancer have been linked to it [24]. A few studies found an increased level of the Hh pathway components in CRC. In addition, in CRC cells in vivo, increased SHH expression was detected at both the mRNA and protein levels. In this regard, several significant genes are associated with the SHH pathway in malignancies of the digestive system. SHH signaling is dormant in the tissues of adult mammals. However, it becomes active during differentiation, proliferation, and maintenance in various adult tissues [25]. It is yet unknown how ATF4 contributes to gastric cancer on a cellular level or whether it stimulates the SHH protein through transcription. These issues are what we work to address.

## 2. Materials and Methods

The GEPIA database provided the differential expression analysis of ATF4 bioinformatics in the neighboring normal tissues and gastric cancer tissues. Eighty pairs of paraffin tissues and four pairs of fresh surgical tumor specimens were utilized as clinical specimens in the experiment and were procured from North Jiangsu People’s Hospital. This study was supported by the Ethics Committee of North Jiangsu People’s Hospital with the ethics number 2019KY-022.

### 2.1. Immunohistochemistry (IHC)

A tissue microarray (TMA) that included samples from 80 patients with histologically confirmed gastric cancer and 80 controls was generated according to a previously described method [26]. The sections were deparaffinized in xylene, rehydrated in a graded alcohol series and citrate buffer, and then blocked with 3% hydrogen peroxide. Subsequently, the sections were incubated with a primary antibody directed against ATF4 (1:100; 11815, Cell Signaling Technology, Danvers, MA, USA) and then with a biotin-conjugated secondary antibody (SA1050; Boster, Wuhan, China), followed by incubation with a streptavidin–peroxidase complex. Five high-power fields (400× magnification) were selected randomly and photographed for each slide. The protein expression scoring was evaluated by taking both the proportion of positive cells (0 (<5%), 1 (5–25%), 2 (26–50%), 3 (51–75%), and 4 (>75%)) and the intensity of cell staining (0 (negative), 1 (weak), 2 (moderate), and 3 (strong)) into account. The final staining scores were calculated by multiplying the staining intensity by the degree of staining. ATF4 staining was considered low or high using a cutoff value of 5 based on the analysis from the receiver operating characteristic (ROC) curve. A final score greater than 5 was defined as a high expression of ATF4.2.2.P.

The GES-1 Normal Gastric Epithelial Cell Line and the GC cell lines, AGS, MGC803, and HGC27, were all developed by Shanghai Gene Chemical Company (Chinese Academy of Science, Shanghai, China). The cells were tested regularly for mycoplasma contamination to ensure they were uncontaminated. MGC803 and HGC27 in RPMI-1640 medium (Invitrogen, Carlsbad, CA, USA) and AGS in F12K (Invitrogen, Carlsbad, CA, USA) and GES1 in DMEM (Invitrogen, Carlsbad, CA, USA) were cultured supplemented with 10% fetal bovine serum (Invitrogen, Carlsbad, CA, USA) and 100 units/mL of penicillin 100 mg/mL streptomycin (Hyclone SV30010) at 37 °C in a humidified incubator containing 5% CO_2_.

### 2.2. Cell Transfection

We employed lentivirus to knock down ATF4 in AGS cells and overexpress ATF4 in the MGC803 cell line to create gastric cancer cell lines with stable ATF4 knockdown or overexpression. Four recombinant lentiviral vectors were constructed: Vector1 (lentivirus-EGFP-Puro), ATF4 (lentivirus-ATF4-EGFP-Puro), Si-ATF4 (lentivirus-EGFP-Si-ATF4-Puro), and Vector2 (lentivirus-EGFP-pRNAi-Puro) from GeneChem (Shanghai, China). In a 24-well plate, 1 × 10^4^ cells were planted into each well 12 h before virus infection. Lentivirus should be added to each well for 72 h, followed by puromycin screening to identify stable cell lines and fluorescence microscopy detection of the fluorescence signal in the cells.

GenePharma (Shanghai, China) provided 1-SHH and pcDNA3.1-NC, the negative control. Twenty-four-well plates were seeded with a total of 1 × 10^4^ cells per well for 12 h, and Lipofectamine^®^ 2000 (Thermo Fisher, Waltham, MA, USA) was used to transfect those cells. Cells that had undergone a two-day transfection were collected for the subsequent studies, utilizing a Weston blot to measure transfection effectiveness.

### 2.3. Western Blot

GC tissues or entire cells were used to extract equivalent amounts of protein, which were then separated with 10% SDS-PAGE gel and electro-transferred onto polyvinylidene fluoride (PVDF) membranes (Thermo Fisher Scientific, Waltham, MA, USA). The antibodies were incubated with various primary antibodies overnight at 4 °C after blocking in 5% milk for 2 h; rabbit anti-ATF4-1 (1:3000, Cell Signaling Technology, Danvers, MA, USA), rabbit anti-SHH (1:3000, Cell Signaling Technology, Danvers, MA, USA), mouse anti-Gli1 (1:3000 Santa Cruz Bicycles, California, CA, USA), rabbit anti-GAPDH (1:5000, Cell Signaling Technology, Danvers, MA, USA), and mouse-anti GAPDH (1:5000, from Abclonal, Wuhan, China) antibodies were used as internal controls. Signals were then detected using an upgraded chemiluminescence substrate after incubating with a secondary antibody bound to peroxidase (Millipore, Schwalbach, Germany). Image J was used to measure the intensity of the bands on the Western blot.

### 2.4. Cell Proliferation Assay

A 96-well plate was filled with 1500 cells in each well and then incubated at 37 °C overnight. Each well received 10 μL of the Cell Counting Kit-8 (CCK-8) (Beyotime Institute of Biotechnology, Shanghai, China), which was then added to the empty media and incubated for two hours at 37 °C. At 24, 48, and 72 h, absorbance was measured at 450 nm using a microplate reader.

### 2.5. Colony Formation

The plate cloning test assessed the cells’ ability to multiply. A 6-well plate was filled with 500 cells per well and cultured for two weeks. The colonies were stained with 0.1% crystal violet after being fixed with 4% paraformaldehyde. We used a digital camera to count the colonies. At least three duplicates of each experiment were carried out.

### 2.6. Cell Migration and Invasion Assays

Transwell with an 8-um pore and Matrigel (Corning Co, Corning, NY, USA) was used to assess the GC cell lines’ capacity for invasion. Using 200 μL of DMEM without FBS indicated 2 × 10^4^ cells per well were planted into the upper chamber after 48 h of transfection. The lower chambers were then filled with 500 μL of the medium, which included 10% FBS, a chemoattractant. After twenty-four hours, the invading cells were preserved and a cotton swab was used to remove any cells left on the upper membrane. Cells that had been invaded were stained with 1% crystal violet after being fixed in 4% formaldehyde. Ten random visual fields were counted using an inverted Nikon microscope. An 8-um pore Transwell insert without Matrigel was used for the migration experiment. The invasion assay was conducted similarly. Wound healing assay and AGS and MGC-803 cells were seeded into 6-well plates and cultured in a complete medium to produce a confluent monolayer. Then, a 200 µL pipette tip was used to scratch a straight wound, and collected tissues were washed three times with PBS to remove debris. Moreover, the medium containing 3% FBS was replaced to culture the remaining cells. Photos were taken at 0 and 24 h after the scratching. Wound closure was evaluated by ImageJ software.

### 2.7. Chromatin Immunoprecipitation (ChIP) Assay

The Simple ChIP Plus Enzymatic Chromatin IP Kit (Magnetic Beads) was used to perform chromatin immunoprecipitation (ChIP) (9005S; Cell Signalling Technology, Danvers, MA, USA). In a nutshell, 3 plates per treatment were utilized to seed 4 × 10^6^ AGS and MGC-803 cells in 150 mm diameter dishes. The Simple ChIP Plus Enzymatic Chromatin IP Kit (Magnetic Beads) instructions (9005S; Cell Signalling Technology, Danvers, MA, USA) were strictly adhered to. Each plate contained 20 mL of medium that had been cross-linked with 1% formaldehyde at room temperature for 10 min. The cross-linking was then stopped by adding 2 mL of glycine, which was harvested in ice-cold PBS containing protease and phosphatase inhibitors after three ice-cold PBS washes per dish. According to the manufacturer’s instructions, chromatin was prepared and dispersed by partial digestion with Micrococcal Nuclease, followed by a mild sonication (Bioruptor Diagenode, Seraing, Belgium). ATF4 rabbit monoclonal antibody (11815, Cell Signaling Technology, Danvers, MA, USA), common rabbit IgG antibody, and ChIP-Grade Protein G Magnetic Beads were used in chromatin immunoprecipitations. Utilizing spin columns, DNA was purified following the reversal of protein–DNA cross-links. DNA was amplified using standard PCR with the addition of a hot-start Taq enzyme Kit (AG11201; Accurate Biology, Changsha, China); the results were detected using agarose electrophoresis. Positive and negative control primers were from the kit: SHH Primer F: GAGGAGTCTCTGCACTACGAG, R: GATATGTGCCTTGGACTCGTAG.

### 2.8. Xenotransplantation Experiment [27]

A total of 10 4-week-old BALB/c male nude mice (weight, 18–22 g) were purchased from GemPharmatech Co. Ltd. (Nanjing, China) and raised in a pathogen-free laminar flow cabinet throughout the experiments under the following conditions: Controlled humidity (30–40%), a constant temperature of 25 °C, a 12-h light/dark cycle and free access to food and water. The ethical approval (approval no. 202111020) to perform the animal experiments was obtained from the Ethics Committee for Animal Experiments of the Yangzhou University (Yangzhou, China). The experimental protocol was performed in accordance with the Laboratory Animal Guideline for Ethical Review of Animal Welfare (26). 4-week-old male BALB/c nude mice were randomly divided into Vector and shATF4 groups (*n* = 5 in both groups). Under isoflurane inhalation anesthesia (1–2%), ~1 × 10^6^ AGS cells of stably transfected strains Vector/shATF4 resuspended in 100 µl PBS were subcutaneously into the left armpit of the mice. The health and behaviour of the mice were monitored every 2 days to determine if there were difficulties eating or drinking, unrelieved pain or distress without recovery. If the tumor reached 2000 mm^3^, the animal would be euthanized as a humane endpoint. The following formula was used to calculate the tumor volume (V) every week: V = (Width^2^ × Length)/2. Four weeks post-inoculation, all the mice were sacrificed by cervical dislocation under anesthesia. The method of anesthesia used for the mice was CO_2_ asphyxiation (CO_2_ was introduced into the chamber at a rate of 40–70% of the chamber volume per min to minimize distress). Dilated pupils were then used to verify death. Then, the tumors were removed and weighed.

### 2.9. Statistical Analyses

The GraphPad Prism 8 program (GraphPad 8.0.1 Software, La Jolla, CA, USA) was used for statistical analysis, and the T-test was used. The correlation between gene expression was carried out using Spearman statistical methods. All data are expressed as the means ± SEM of at least three independent experiments; * *p* < 0.05, ** *p* < 0.01, *** *p* < 0.001, **** *p* < 0.0001 are considered significant.

## 3. Results

### 3.1. The Expression of ATF4 Is Increased in Gastric Cancer Tissues and Gastric Cancer Cell Lines

**Figure 1:**
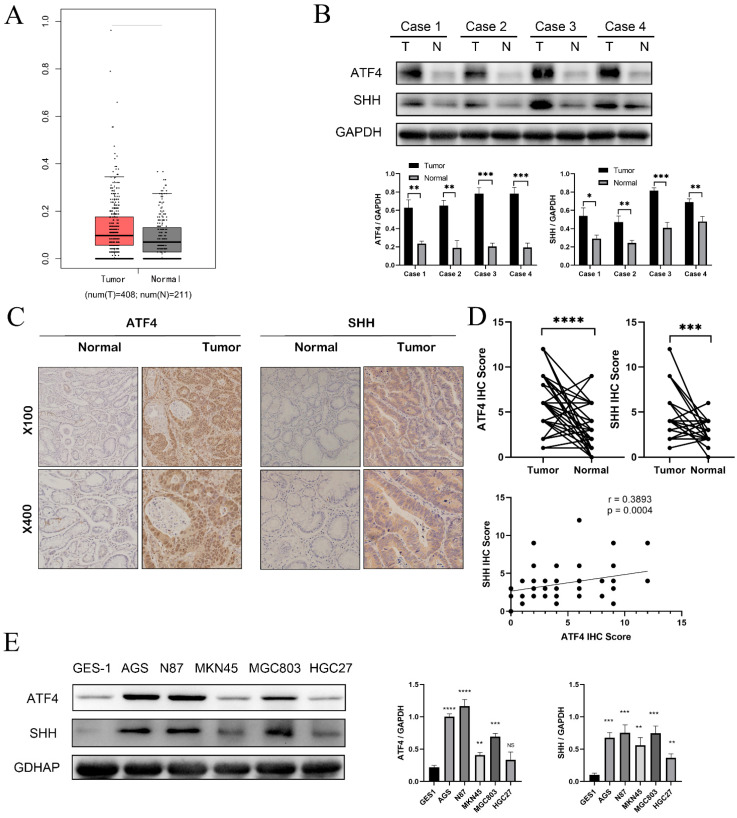
ATF4 is highly expressed in gastric cancer tissues and gastric cancer cell lines. (**A**) The expression of ATF4 is higher in gastric cancer (*n* = 408) than in normal samples (*n* = 211) in the GEPIA database. (**B**) Western blot analysis results of ATF4 in four pairs of gastric and adjacent gastric tissue. (**C**) Immunohistochemical analysis of ATF4 and SHH expression in a GC patient tissue array (*n* = 80). Representative images are shown. (**D**) Paired t-test showing results of immunohistochemistry. (**E**) Western blot shows the expression of ATF4 in human gastric mucosal epithelial cells (GES-1) and gastric cancer cell lines. * *p* < 0.05, ** *p* < 0.01, *** *p* < 0.001, and **** *p* < 0.0001. NS means statistically insignificant. The original western blot figures could be found in Supplementary S2.

We initially identified the expression of ATF4 in gastric and surrounding tissues in the GEPIA database (http://gepia.cancer-pku.cn/ (accessed on 15 May 2022)) to evaluate the role of ATF4 in the pathogenesis of gastric cancer. ATF4 was considerably higher in gastric cancer than in surrounding tissues when we examined 408 gastric tumors and 211 normal tissues from the GEPIA database (Figure 1A). The expression of ATF4 and SHH was then found in four pairs of fresh gastric cancer tumor tissues. Fresh tumor tissue from gastric cancer showed similar outcomes (Figure 1B). We used immunohistochemical (IHC) analysis to examine the expression of ATF4 in 80 pairs of gastric cancer tissues and nearby normal tissues to better understand how ATF4 is expressed in gastric cancer. Compared to the surrounding non-cancerous tissues, the expression of ATF4 and SHH in stomach cancer tissues was greater, and the correlation between ATF4 and SHH was detected in 80 clinical gastric cancer tissues by immunohistochemistry score. There is a positive correlation between ATF4 and SHH protein expression. The results are presented in Figure 1C. It is statistically significant that gastric cancer has a high expression of ATF4 (Figure 1D). ATF4 and SHH are significantly expressed in gastric cancer cell lines, as seen in our examination of its expression in Human Gastric Mucosal Epithelial Cells (GES1) (Figure 1E).

### 3.2. ATF4 Knockdown Strongly Inhibits the Proliferation and Invasion of GC Cells

**Figure 2:**
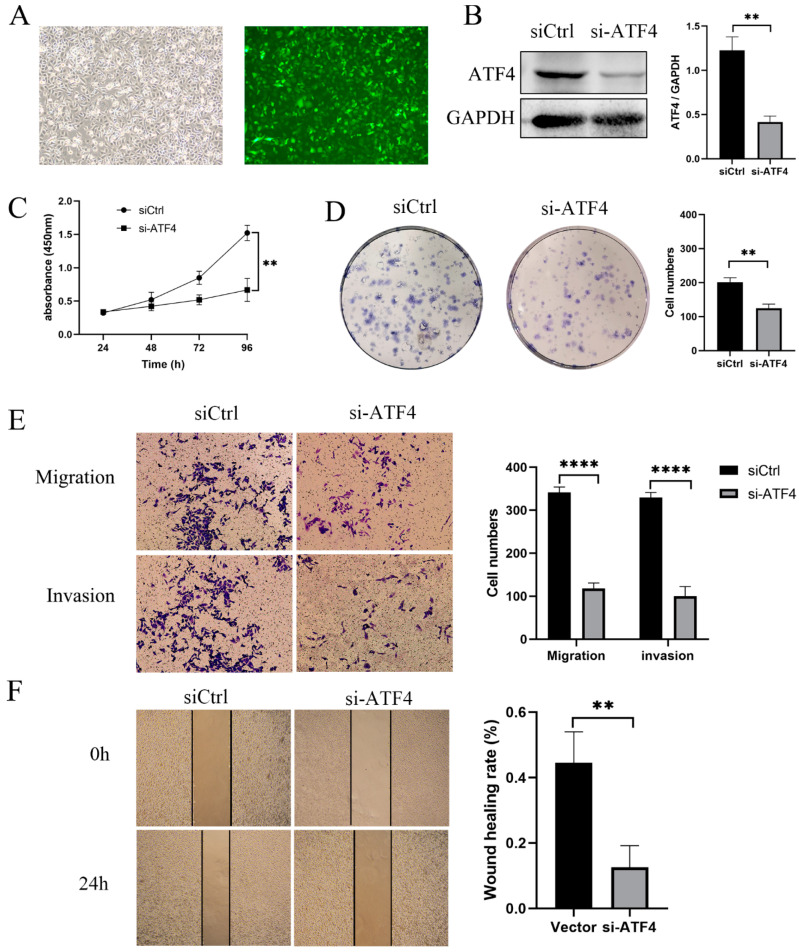
ATF4 knockdown strongly inhibits the proliferation and invasion of GC cells. (**A**) The lentiviral vector contains the green fluorescent protein sequence, and successfully transfected cells will express the green fluorescent protein. (**B**) Compared with cells transfected with empty vector, the expression of ATF4 in cells transfected with knockdown of ATF4 was significantly reduced. (**C**,**D**) CCK8 and colony formation assay were used to assess whether ATF4 affects the proliferation of the AGS cell. The results showed that compared with the control group, the proliferation rate of cells after knocking down ATF4 was significantly reduced. (**E**,**F**) MGC803 cells overexpressing ATF4 have improved abilities for migration and invasion, (**E**) light microscopy (×20), (**F**) light microscopy (×10). ** *p* < 0.01, and **** *p* < 0.0001. The original western blot figures could be found in Supplementary S2.

The increased expression of ATF4 in gastric cancer has been verified to assess ATF4’s contribution to the emergence of gastric cancer. To reduce endogenous ATF4 in AGS, lentivirus-mediated ATF4-specific short hairpin RNAs (shRNAs) were transfected. The green fluorescent protein sequence is present in the lentiviral vector, and cells that have been successfully transfected will produce the protein (Figure 2A). After puromycin selection, stable ATF4 knockdown cells were produced. The expression of ATF4 was dramatically reduced in cells transfected with the knockdown of ATF4 compared to cells transfected with an empty vector (Figure 2B). In order to determine whether ATF4 has an impact on the proliferation of AGS cells, a CCK8 and colony formation test were performed. The findings demonstrated that knocking down ATF4 considerably decreased the proliferation rate of cells in comparison to the control group (Figure 2C,D). After that, we looked at how AGS cells migrated and invaded after having ATF4 knocked down. The findings of transwell and wound-healing assays revealed that the migration in the sh-ATF4 group compared with the Vector group in AGS cell was dramatically reduced (Figure 2E,F). The transwell with Matrigel results revealed that the sh-ATF4 group’s capacity for invasion was decreased (Figure 2E). The sh-ATF4 group was compared with the growth, migration, and invasion capacities of the control and siRNA treated AGS with HGC27 cell lines naturally under-expressing ATF4. There was no significant difference in growth, migration, and invasion capacity between the sh-ATF4 AGS group and the HGC27 group. These results are presented in Supplementary S1A,B.

### 3.3. ATF4 Upregulation Promotes the Proliferation and Invasion of GC Cells

**Figure 3:**
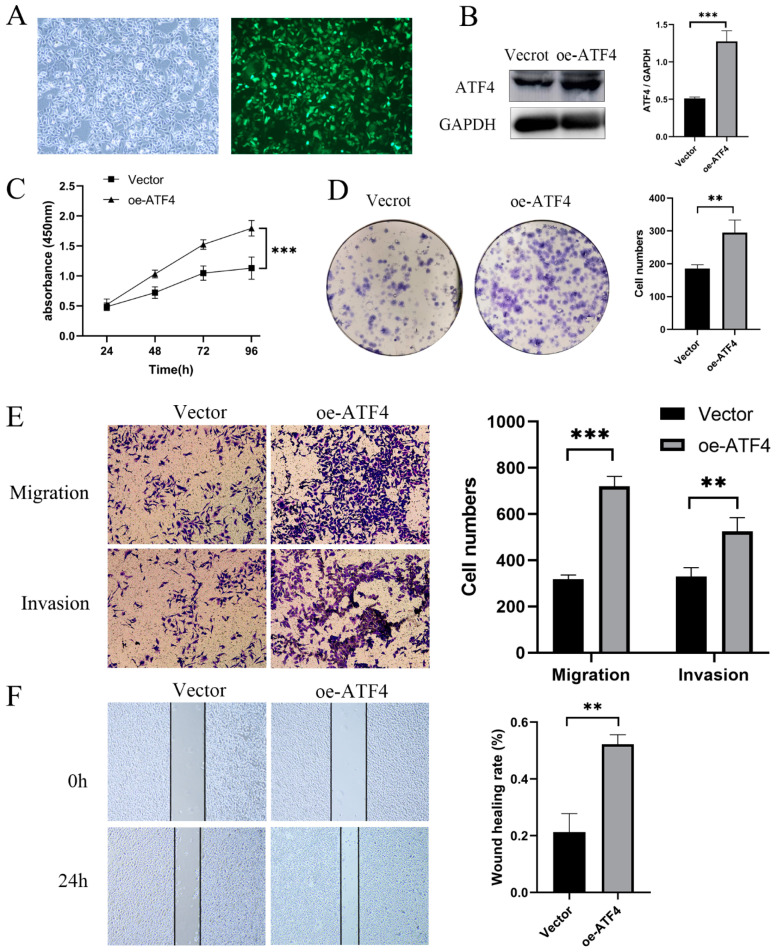
ATF4 upregulation promotes the proliferation and invasion of GC cells. (**A**) The lentiviral vector contains the green fluorescent protein sequence, and successfully transfected cells will express the green fluorescent protein. (**B**) Compared with cells transfected with empty vector, the expression of ATF4 in cells transfected with upregulation of ATF4 was significantly increased. (**C**,**D**) CCK8 and colony formation assay were used to assess whether ATF4 affects the proliferation of AGS cell. The results showed that compared with the control group, the proliferation rate of cells after up-regulating ATF4 was significantly increased. (**E**,**F**) The results of transwell and wound-healing experiments showed that the migration was significantly increased in the oe-ATF4 group compared with the Vector group in the AGS cell, (**E**) light microscopy (×20), (**F**) light microscopy (×10). ** *p* < 0.01, *** *p* < 0.001. The original western blot figures could be found in Supplementary S2.

To determine if ATF4 overexpression promotes gastric cancer cell proliferation and invasion, lentiviral transfection was used to create MGC803 cells that are continuously overexpressing ATF4 (Figure 3A,B). CCK8 and plate clone data demonstrate that ATF4 overexpression can encourage the proliferation of AGS cells (Figure 3C,D). Additionally, as seen in Figure 3E,F, MGC803 cells overexpressing ATF4 have improved abilities for migration and invasion. There was no significant difference in growth, migration, and invasion capacity between the oe-ATF4 MGC803 group and AGS group (Supplementary S1C,D).

### 3.4. Transcription Factor ATF4 binds to the Promoter Region of SHH to Activate the Sonic Hedgehog Pathway

**Figure 4:**
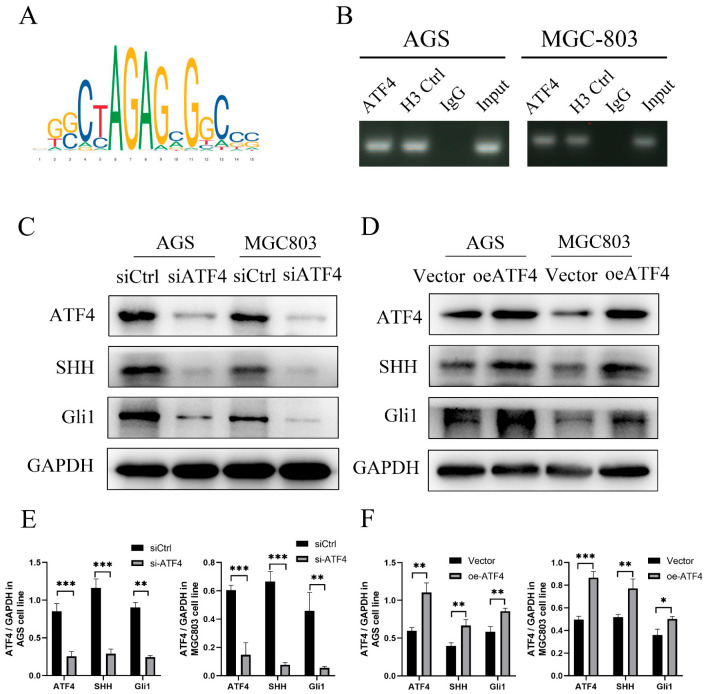
ATF4 binds to the promoter region of SHH to activate the Sonic Hedgehog pathway. (**A**) ATF4 is predicted to be able to bind to the SHH promoter sequence in the JASPA database. (**B**) ATF4 recruitment to the SHH promoter was further demonstrated by the findings of chromatin immunoprecipitation (ChIP) tests in AGS and MGC803. (**C**) Knockdown of ATF4 dramatically decreased SHH protein and downstream Gli1 protein in the Sonic Hedgehog pathway in the AGS and MGC-803. (**D**) The AGS and MGC-803 cell lines greatly boosted the expression of SHH and GLI proteins when we overexpressed ATF4. (**E**,**F**) Quantification of Western blot analysis by Image J software. * *p* < 0.05, ** *p* < 0.01, and *** *p* < 0.001. The original western blot figures could be found in Supplementary S2.

The transcription factor ATF4 is predicted to be able to bind to the SHH promoter sequence in the JASPA database that predicts transcription factor binding sites (https://jaspar.genereg.net/ (accessed on 20 May 2022)), and the predicted binding site is GGCTAGAGCGGCCC (Figure 4A). We created primers for the SHH promoter region to confirm the anticipated results. ATF4 recruitment to the SHH promoter was further demonstrated by the findings of chromatin immunoprecipitation (ChIP) tests in AGS and MGC-803 (Figure 4B). We treated AGS, MGC-803 with ATF4 siRNA and ATF4 overexpression to ascertain if ATF4 operates upstream of SHH in GC. Intriguingly, in the AGS and MGC-803 cell line, the knockdown of ATF4 dramatically decreased SHH protein and downstream Gli1 protein in the Sonic Hedgehog pathway (Figure 4C).

The AGS and MGC-803 greatly boosted the expression of SHH and GLI proteins when we overexpressed ATF4 (Figure 4D). Quantification of Western blot analysis by Image J software (Figure 4E,F). ATF4 binding to the UHMK1 promoter in GC cell lines was further demonstrated by the findings of chromatin immunoprecipitation (ChIP) tests. Consequently, ATF4 encourages SHH transcription in GC, creating a positive feedback loop.

### 3.5. ATF4 Regulates the Proliferation and Invasive Ability of Gastric Cancer Cells through SHH

**Figure 5:**
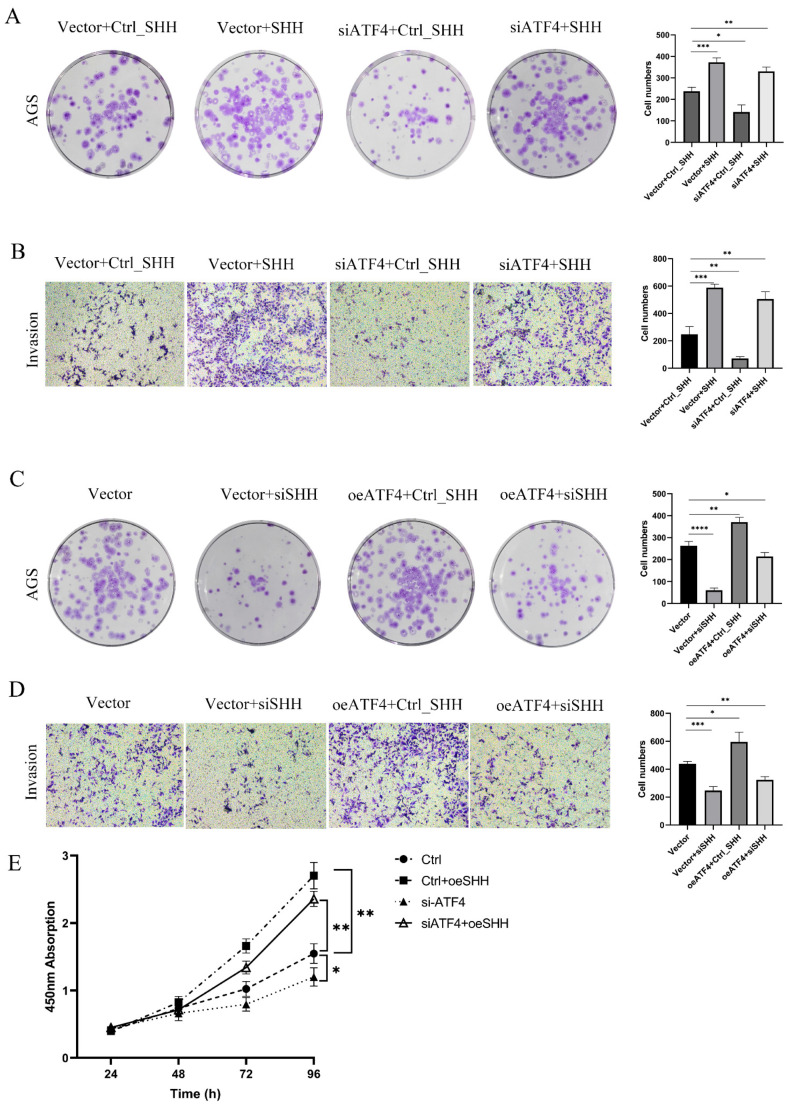
ATF4 regulates the proliferation and invasive ability of gastric cancer cells through SHH. (**A**) After SHH overexpression, the proliferative capacity of AGS knockdown ATF4 cells was nearly restored to its initial level as compared to the group transfected with si-ATF4 lentivirus and pcDNA 3.1. (**B**) The invasive potential of AGS cells produced comparable outcomes, light microscopy (×20). (**C**) SHH protein expression was decreased in MGC-803 cells that were stably overexpressing ATF4, and this dramatically decreased the cells’ ability to proliferate. (**D**) MGC803 cells that were stably overexpressing ATF4 had considerably less ability to invade after SHH expression was knocked down, light microscopy (×20). (**E**) CCK8 studies demonstrate that reversing SHH expression considerably improves colony-forming capacity. * *p* < 0.05, ** *p* < 0.01, *** *p* < 0.001, and **** *p* < 0.0001.

We devised a series of rescue studies to show whether ATF4 controls the proliferation and invasion capacity of gastric cancer cell lines through SHH protein. pcDNA 3.1-SHH and the si-ATF4 lentivirus were co-transfected into AGS cells. After SHH overexpression, the proliferative capacity of AGS knockdown ATF4 cells was nearly restored to its initial level as compared to the group transfected with si-ATF4 lentivirus and pcDNA 3.1 (Figure 5A). Additionally, the invasive potential of AGS cells produced comparable outcomes (Figure 5B). We simultaneously transfected siSHH and oe-ATF4 lentivirus in MGC-803 cells. As seen in Figure 5C, SHH protein expression was decreased in MGC-803 cells that were stably overexpressing ATF4, and this dramatically decreased the cells’ ability to proliferate. When SHH expression is knocked down in MGC803 cells using an empty vector, the cells’ capacity to proliferate is drastically diminished. According to data from a study on cell invasion, MGC803 cells that were stably overexpressing ATF4 had considerably less ability to invade after SHH expression was knocked down (Figure 5D). In addition to these, CCK8 studies demonstrate that reversing SHH expression considerably improves colony-forming capacity (Figure 5E). The findings above imply that ATF4 may control gastric cancer cell invasion and proliferation through changing the SHH protein.

### 3.6. ATF4 Enhanced Tumor Formation of Gastric Cancer Cells in a Xenograft Model

**Figure 6:**
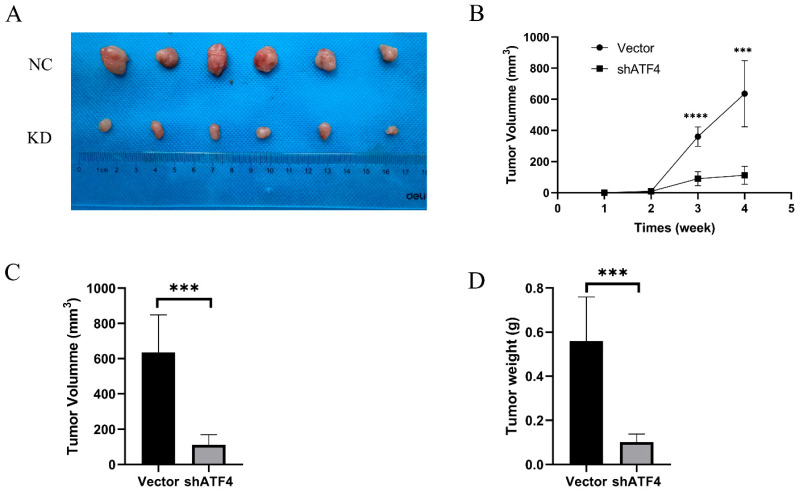
ATF4 knockdown downregulates the tumor formation of gastric cancer cells in a xenograft model. (**A**) Xenograft models in nude mice were generated using AGS cells transfected with Ctrl (*n* = 6) or sh-ATF4 lentiviral (*n* = 6). (**B**) The silencing of ATF4 greatly reduced the growth of the AGS tumor in mice when compared to the control group. (**C**) The average volume of the shATF4 group was considerably smaller than that of the shCtrl group. (**D**) The average weight of the shATF4 group was significantly lower than that of the shCtrl group. *** *p* < 0.001, and **** *p* < 0.0001.

Immunodeficient BALB/c mice bearing AGS cells that had been stably transfected with the vector or sh-ATF4 lentivirus were utilized to determine the involvement of ATF4 in the carcinogenesis of gastric cancer in vivo in order to confirm further whether ATF4 functions in in vivo models. Nude mice aged 5 weeks were subcutaneously injected with AGS NC cells and AGS KD cells. A palpable mass appeared at the injection site one week following the injection. After 4 weeks, the tumors were obtained and examined (Figure 6A). The mass’s largest and smallest diameters were then measured weekly. As anticipated, beginning in the third week, the silencing of ATF4 greatly reduced the growth of the AGS tumor in mice when compared to the control group (Figure 6B). After the fourth week, the nude mice were slaughtered, and tumor weights and volumes were measured. The average volume of the shATF4 group was considerably smaller than that of the shCtrl group (Figure 6C, *p* < 0.0001) (111.8 ± 23.66 vs. 636.3 ± 86.70 mm^3^). The average weight of the shATF4 group (0.1017 ± 0.01493 g) was significantly lower than that of the shCtrl group (0.5600 ± 0.08145 g) (Figure 6D, *p* < 0.0001). The outcomes showed that suppressing ATF4 prevented stomach cancer.

## 4. Discussion

According to reports, ATF4 is crucial in ER-negative breast malignancies, lung cancer, colorectal cancer, prostate cancer, and other types of cancer. ATF4 is highly expressed in triple-negative breast cancer, and ATF4 could promote breast cancer cell proliferation. In triple-negative breast cancer, a high ATF4 expression was shown to be correlated with low OS after diagnosis (37 months for high ATF4 expression and 46 months for low ATF4 expression) [28]. According to previous studies, higher nuclear ATF4 expression was detected in lung cancer cells compared to cytoplasmic ATF4 expression. Lung cancer cells overexpress ATF4 and localize it primarily to the nucleus, which leads to an increase in lung cancer cell proliferation and invasion [29]. Studies have reported that the transcriptional activation of ATF4 promotes colorectal cancer proliferation. ATF4 is a downstream target of URB1 and is involved in the oncogenic role of URB1 in colorectal cancer [30].Our study demonstrated, for the first time, how transcriptional control of the SHH protein by ATF4 affects the development of gastric cancer. In order to stimulate the expression of the SHH protein, which activates the Sonic Hedgehog signaling pathway, ATF4 binds to the SHH promoter region, as our study demonstrated for the first time. By comparing gastric cancer tissues and cells to healthy control tissues and cells, we were able to show that ATF4 is considerably increased in gastric cancer tissues and cancer cells. ATF4 positively regulated the proliferation, invasion, and migration of gastric cancer cells. Furthermore, ATF4 regulates the proliferation, invasion, and migration ability of gastric cancer cells through transcriptional activation of SHH.

Cancer cells usually have an increased level of the stress-induced transcription protein activating transcription factor 4 (ATF4). ATF4 is overexpressed in ER-negative breast tumors and regulates the GSK3/-catenin/cyclin D1 pathway to advance the cell cycle. During glutaminolysis inhibition in colorectal cancer, activating transcription factor 4 (ATF4) is increased to reduce mTOR signaling by transcriptionally activating the mTOR suppressor DNA damage-inducible transcript 4 (DDIT4) [31]. ATF4 is strongly linked to autophagy and the mTORC1 pathway, which Yongxiang Li et al. found to enhance the growth of gastric cancer [32]. The direct interaction between SLFN5 and ATF4 in prostate cancer results in mTOR activation. ATF4 is not identified to regulate SHH, inhibiting the Sonic Hedgehog signaling pathway and preventing the onset and spread of gastric cancer.

The development of the central nervous system during fetal development is mostly correlated with the Sonic Hedgehog (SHH) pathway, an evolutionary conserved molecular cascade. The ligands of the SHH signaling pathway control the activation of this pathway. Gli1 is a marker of Shh pathway activation. As a target gene of the Shh pathway and as a transcription activator downstream of Shh signaling, Gli1 autoregulates and increases Shh signaling output. Our design leaves something to be desired; tumor growth in nude mice could be studied with or without the treatment of animals using a Gli1 inhibitor; for example, GANT61. Such an experiment could bring strong evidence for the importance of developing anticancer therapies targeting the ATF4/SHH pathway. Numerous malignancies, such as retinoblastoma, breast, colorectal, and non-small cell lung cancer, are frequently linked to the growth of SHH pathway components, especially GLI transcription factors [33,34]. SHH was found to be effective in reducing tumor size and angiogenesis in a mouse model of pancreatic cancer [35]. One important molecule that turns on SHH signaling is the SHH ligand. Therefore, it should come as no surprise that research has been conducted on the upstream control of the SHH gene to fully comprehend the significance of the SHH pathway in carcinogenesis [36]. In a prior study, the KRAS proto-oncogene of the MAPK/ERK pathway was found to boost the transcriptional activity of GLI1 and the expression of SHH pathway target genes in gastric cancer [37]. The Hedgehog pathway is one of the most common signal transduction pathways used by mammalian cells. Most studies have focused on its role during development, primarily of the nervous system, skin, bone, and pancreas. SHH plays a significant role during epithelial development and differentiation, homeostasis, and neoplastic transformation of the stomach. Significant levels of Sonic Hedgehog are expressed in the gastric mucosa, which has served to direct analysis of its role during organogenesis, gastric acid secretion, and neoplastic transformation [38,39,40]. Due to the activation of this pathway during proliferation and neoplastic transformation, more recent studies have examined its role in adult tissues.

In summary, we found that ATF4 binds to the promoter region of SHH to activate the Sonic Hedgehog pathway for the first time. The biological function of ATF4 in vivo and in vitro by activating SHH has been established, and a more detailed molecular mechanism of ATF4 and SHH regulating the occurrence of gastric cancer should be investigated in the future.

## 5. Conclusions

We identify ATF4 as a key protein in mediating proliferation, invasion, and migration. This study provides a new understanding of the critical underlying mechanism of ATF4 leading to the proliferation, invasion, and migration of gastric cancer cells through transcriptionally activating SHH.

## Data Availability

The data presented in this study are available on reasonable request.

## Supplementary Materials

The following supporting information can be downloaded at: https://www.mdpi.com/article/10.3390/cancers15051429/s1, Supplementary S1: ATF4 promotes proliferation, invasion, and migration of gastric cancer cells; Supplementary S2: The original western blot figures.
