# Supplementary material for: ATF4 Transcriptionally Activates SHH to Promote Proliferation, Invasion, and Migration of Gastric Cancer Cells
Source: Cancers (Basel). 2023 Feb 23;15(5):1429. doi: 10.3390/cancers15051429 (PMC10000907; doi:10.3390/cancers15051429)
Supplement: Supplementary file 1 [file cancers-15-01429-s001.zip › cancers-2077317-supplementary.pdf]

Supplementary S1

ATF4 promotes proliferation, invasion, and migration of gastric cancer cells.

(Figure AB) ATF4 knockdown strongly inhibits the proliferation and invasion of GC cells, there was no significant difference in growth, migration and invasion capacity between the sh-ATF4 AGS group and the HGC27 group.

(Figure CD) there was no significant difference in growth, migration and invasion capacity between the oe-ATF4 MGC803 group and AGS group.

** $p < 0.01$, *** $p < 0.001$, and **** $p < 0.0001$. ns means statistically insignificant.

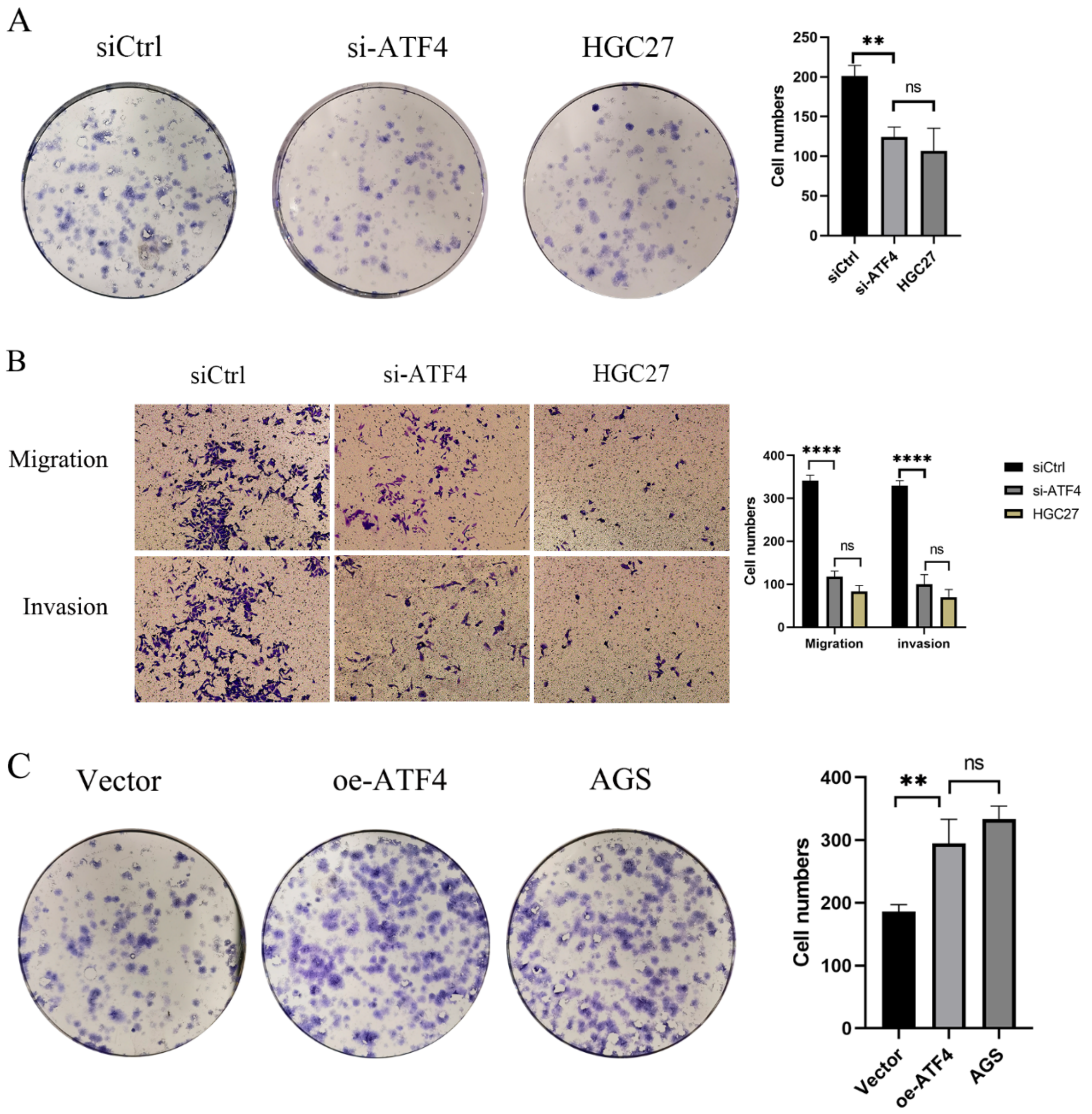

D

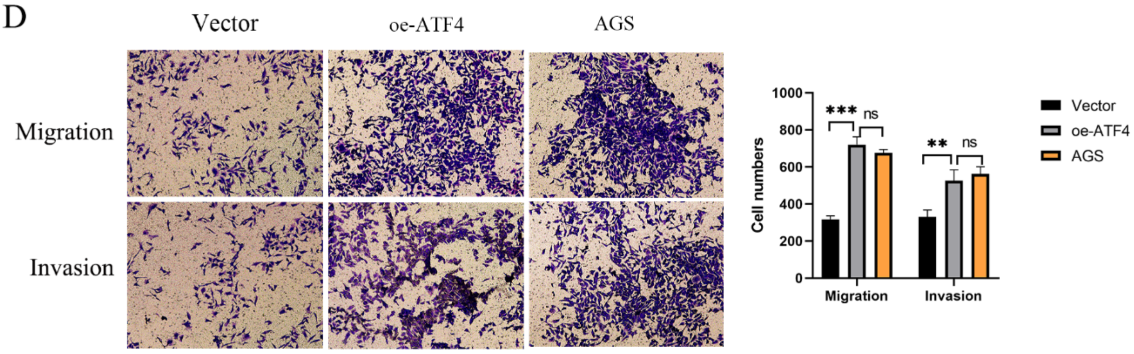

Supplementary S2

The original western blot figures.

Figure 1B

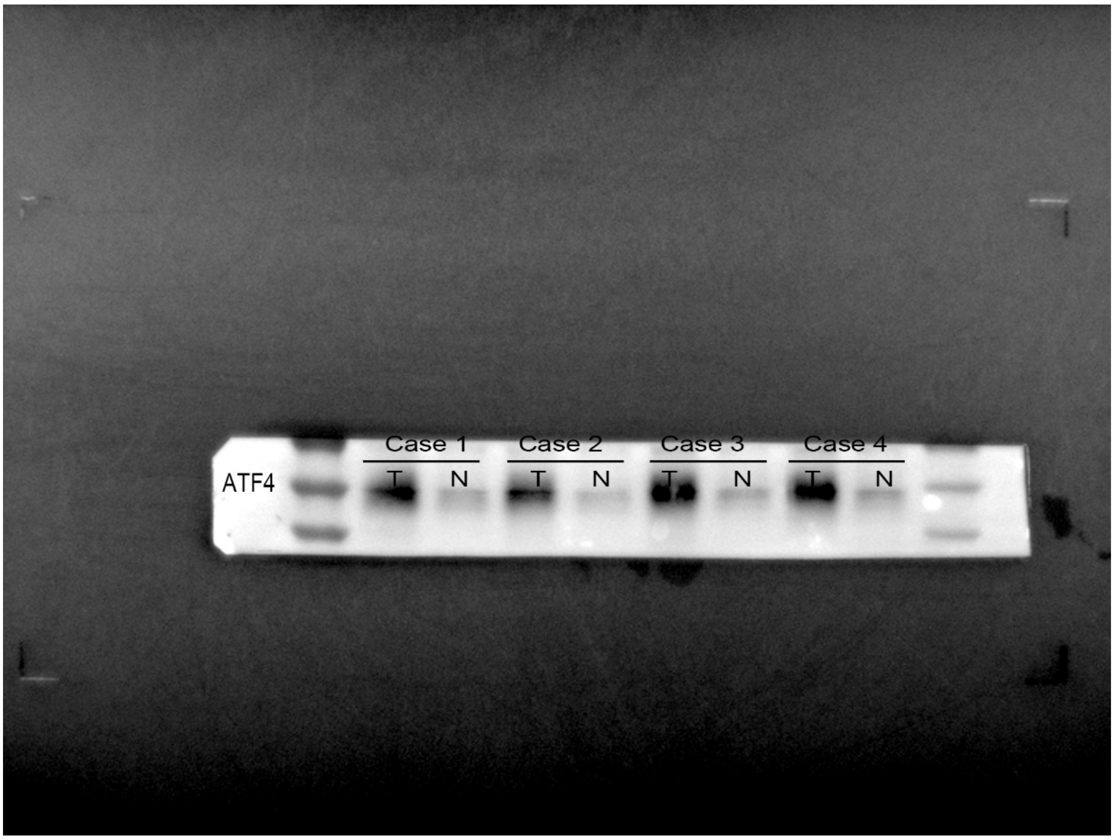

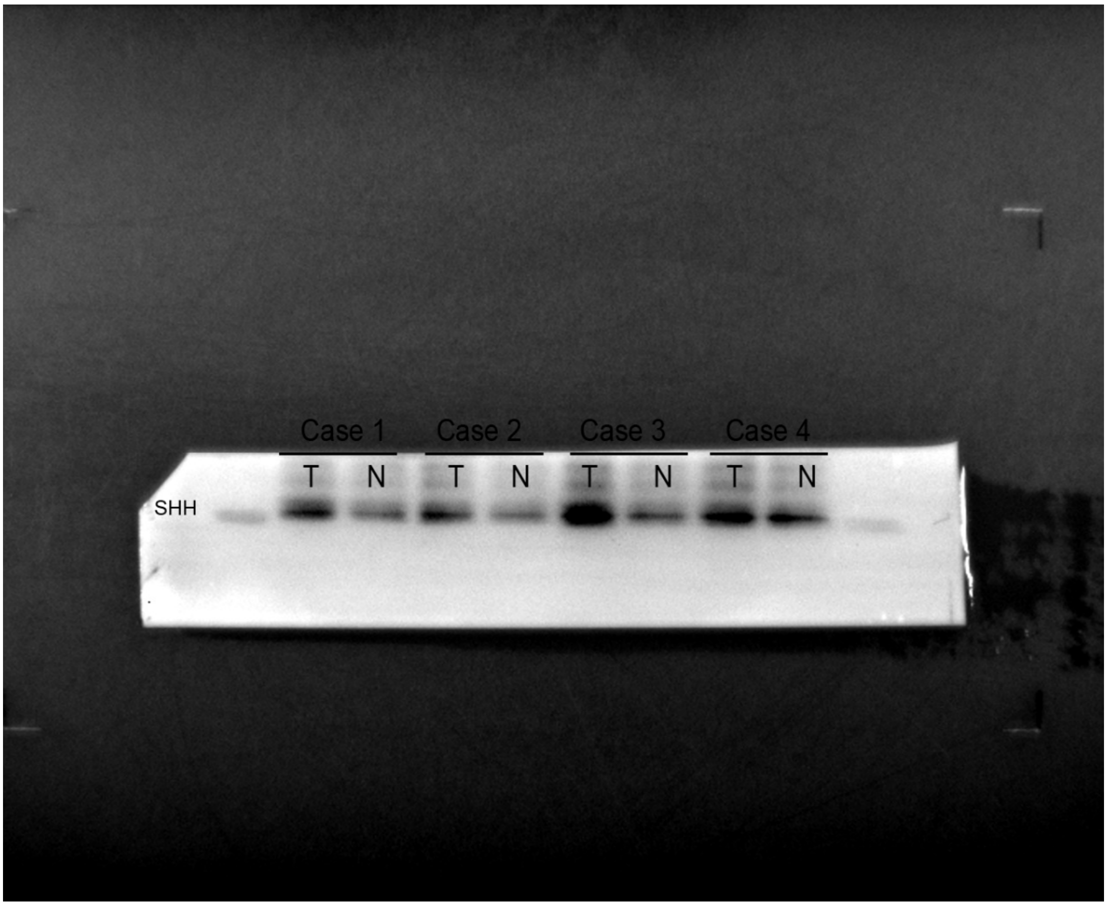
Case 1
Case 2
Case 3
Case 4
T N T N T N T N
SHH

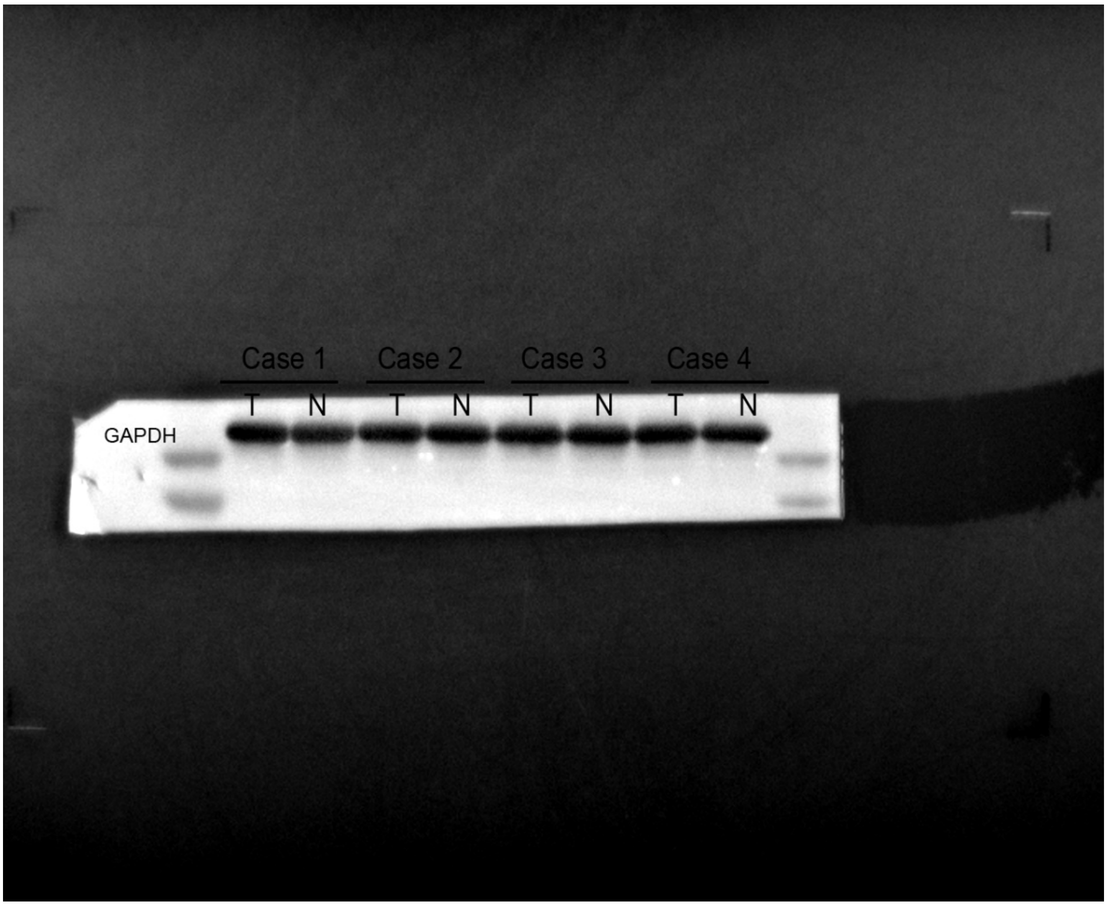
Case 1
Case 2
Case 3
Case 4
T N T N T N T N
GAPDH

Figure 1E

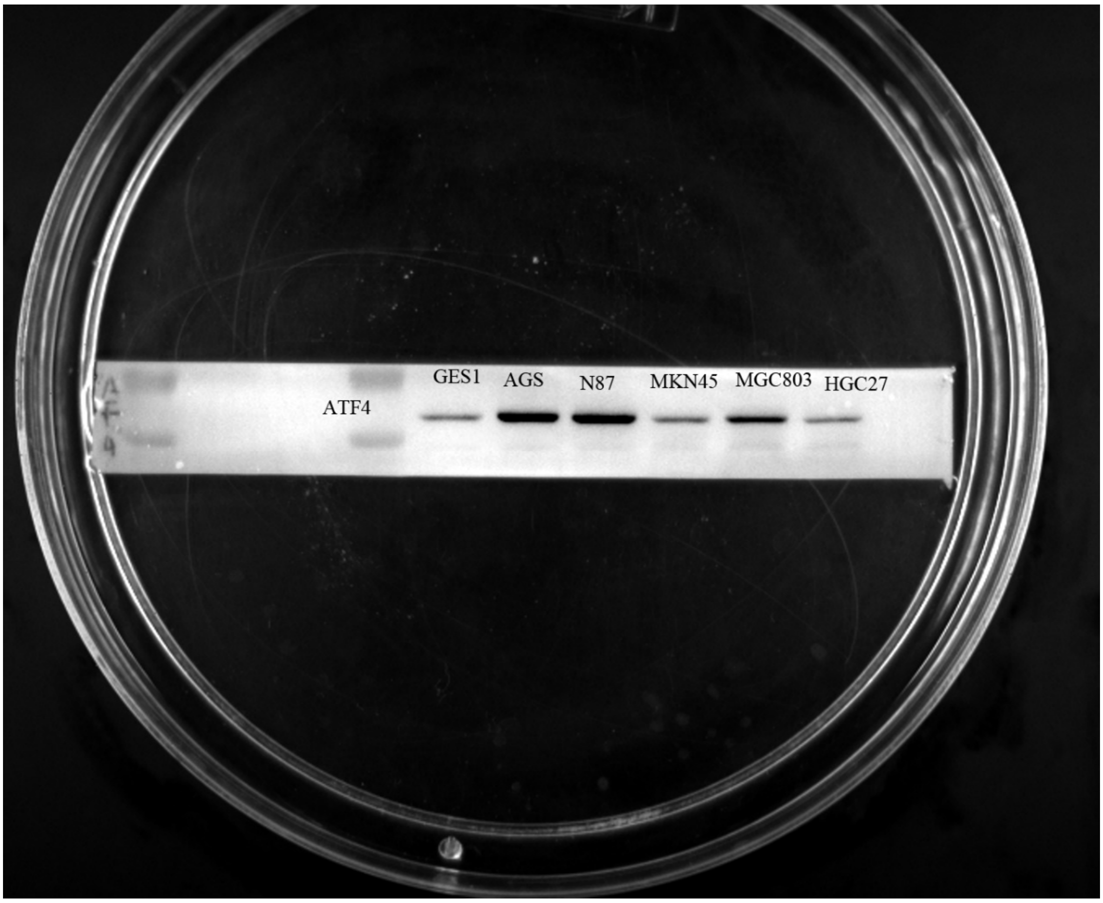

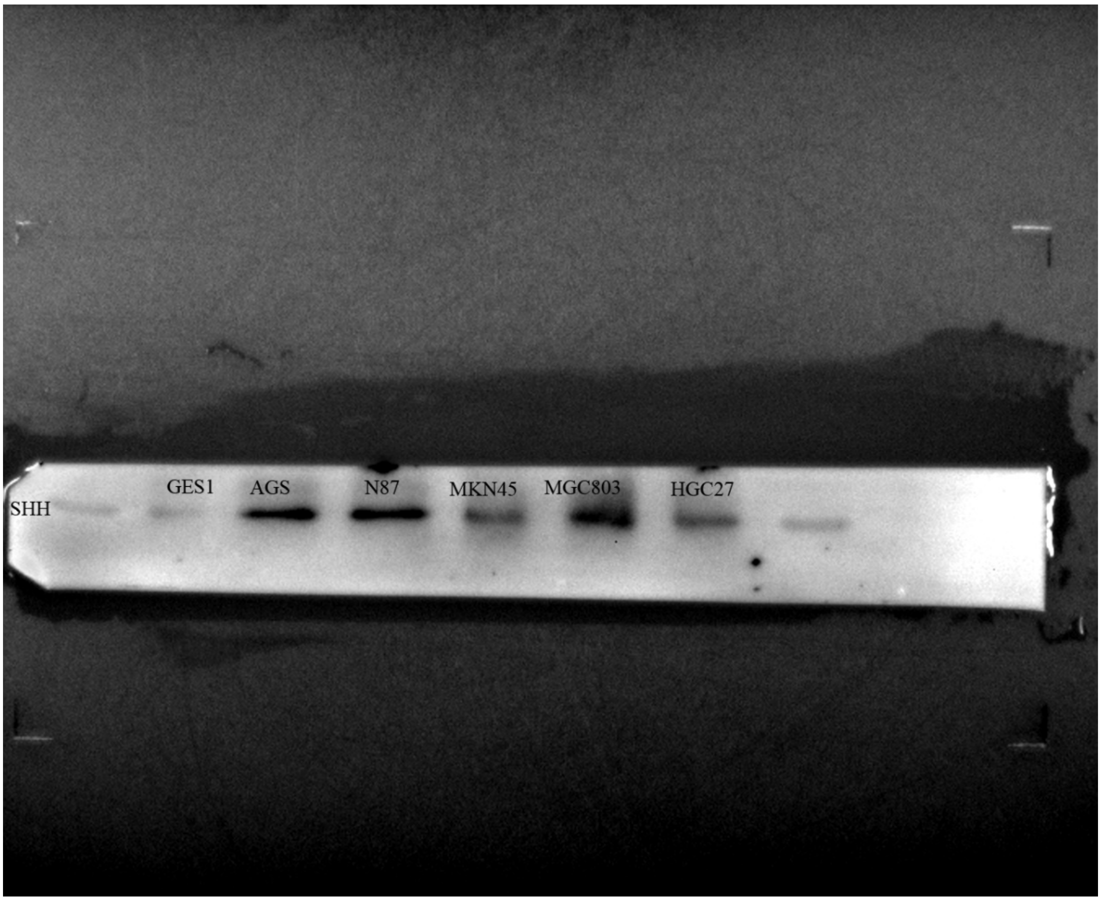
SHH
GES1
AGS
N87
MKN45
MGC803
HGC27

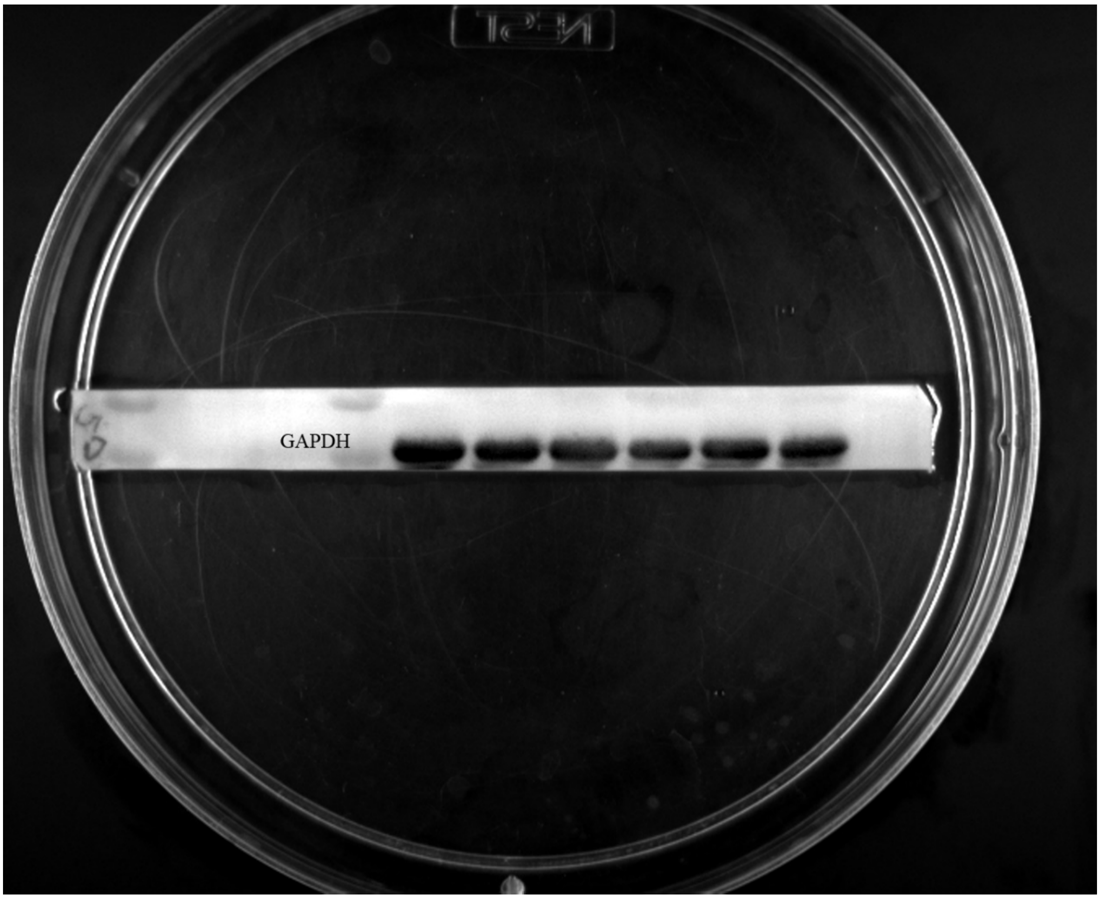
GAPDH

Figure 2B

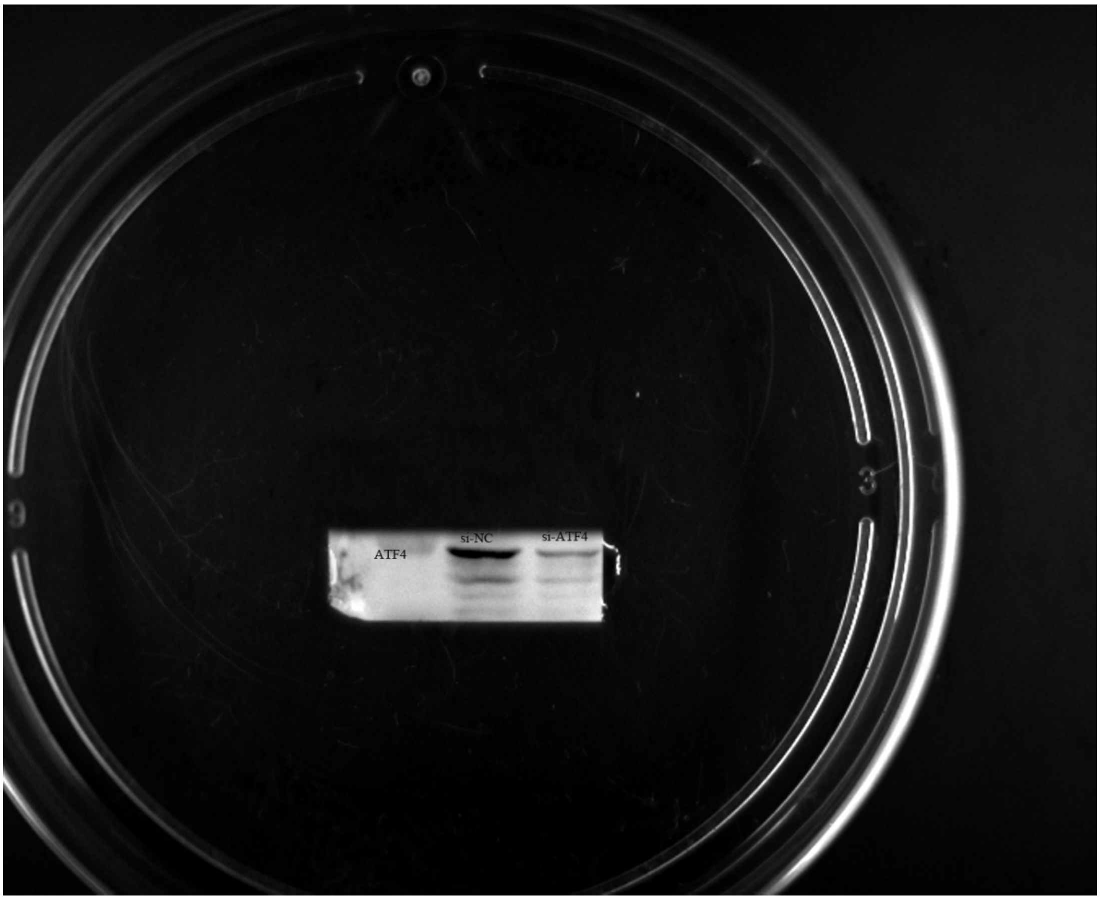

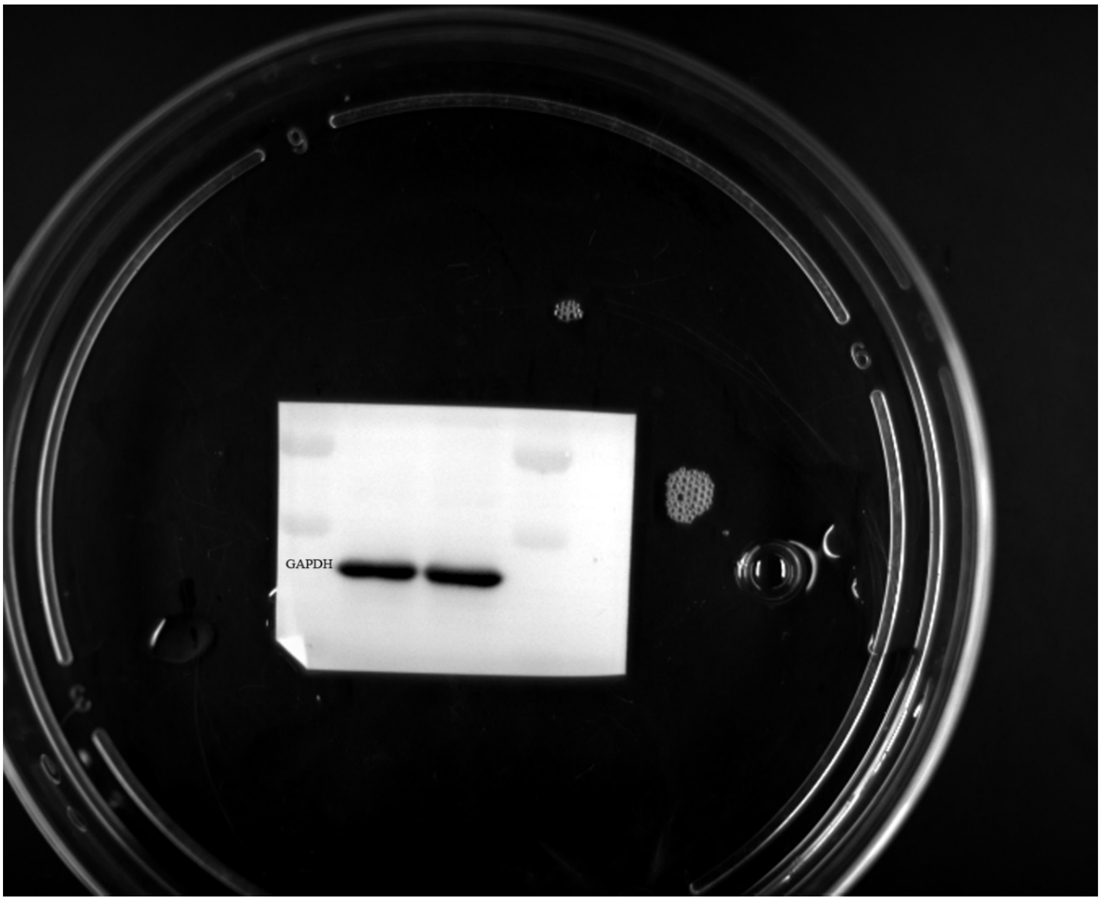
GAPDH

Figure 3B

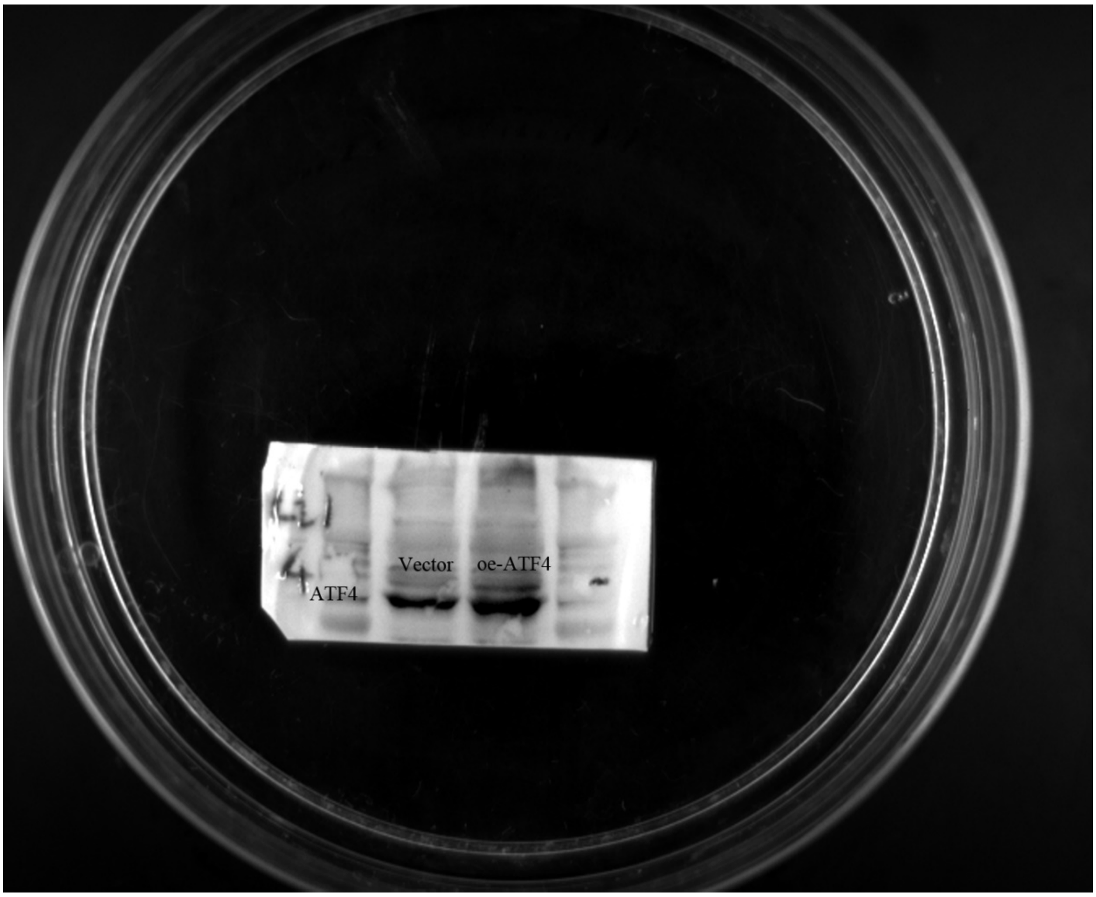

Figure 4C

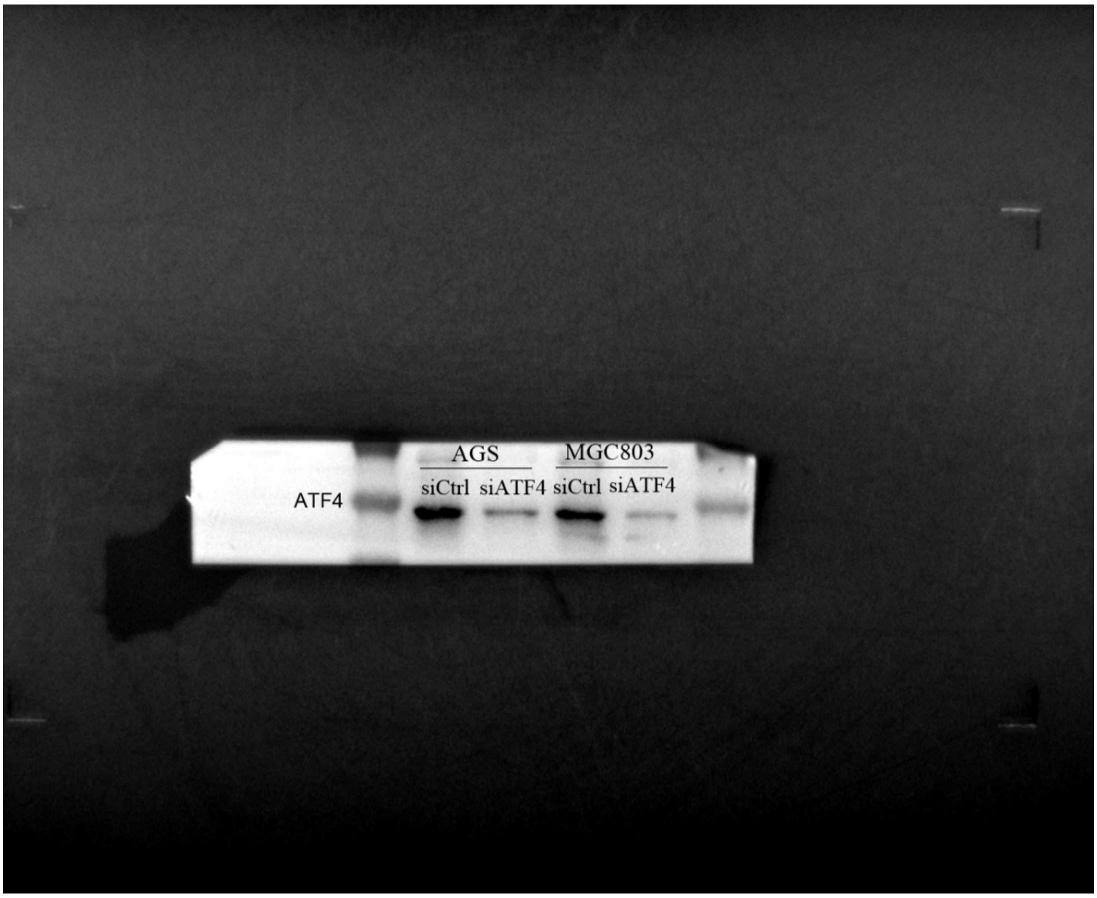

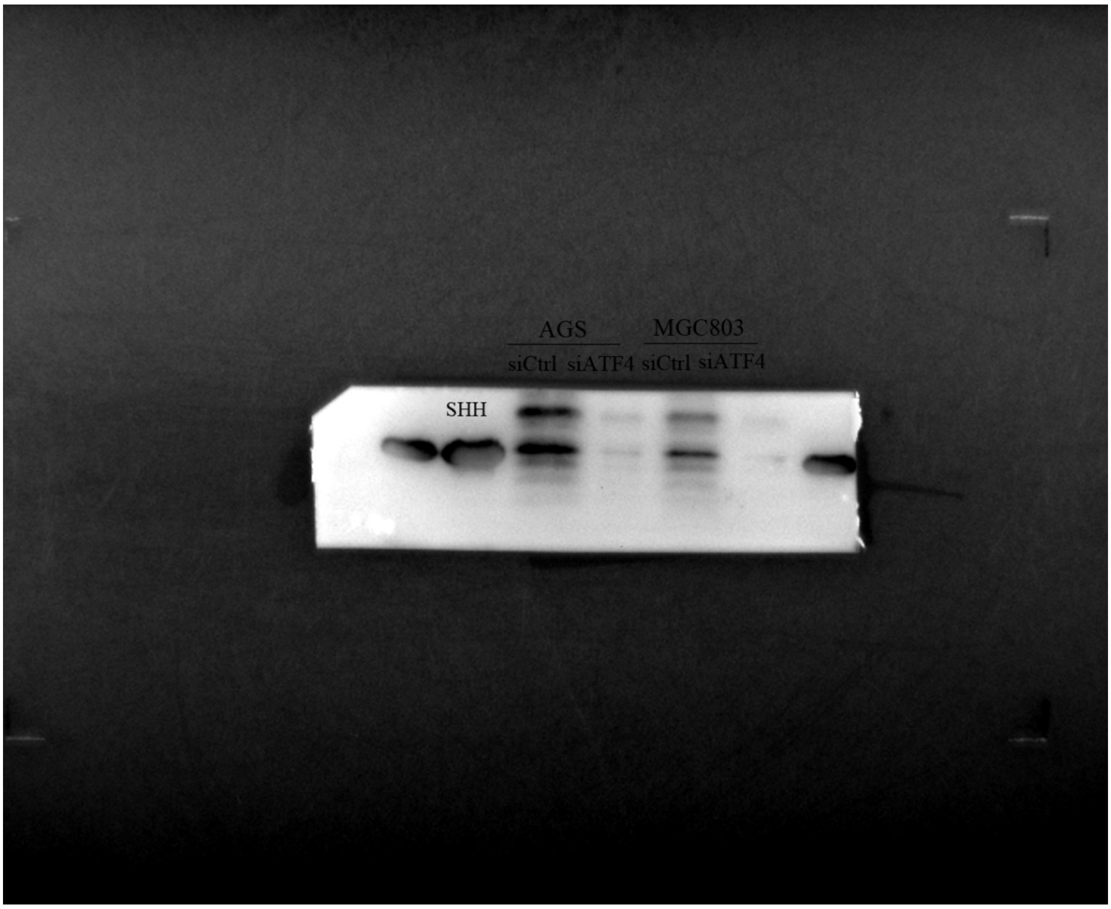
AGS
MGC803
siCtrl siATF4 siCtrl siATF4
SHH

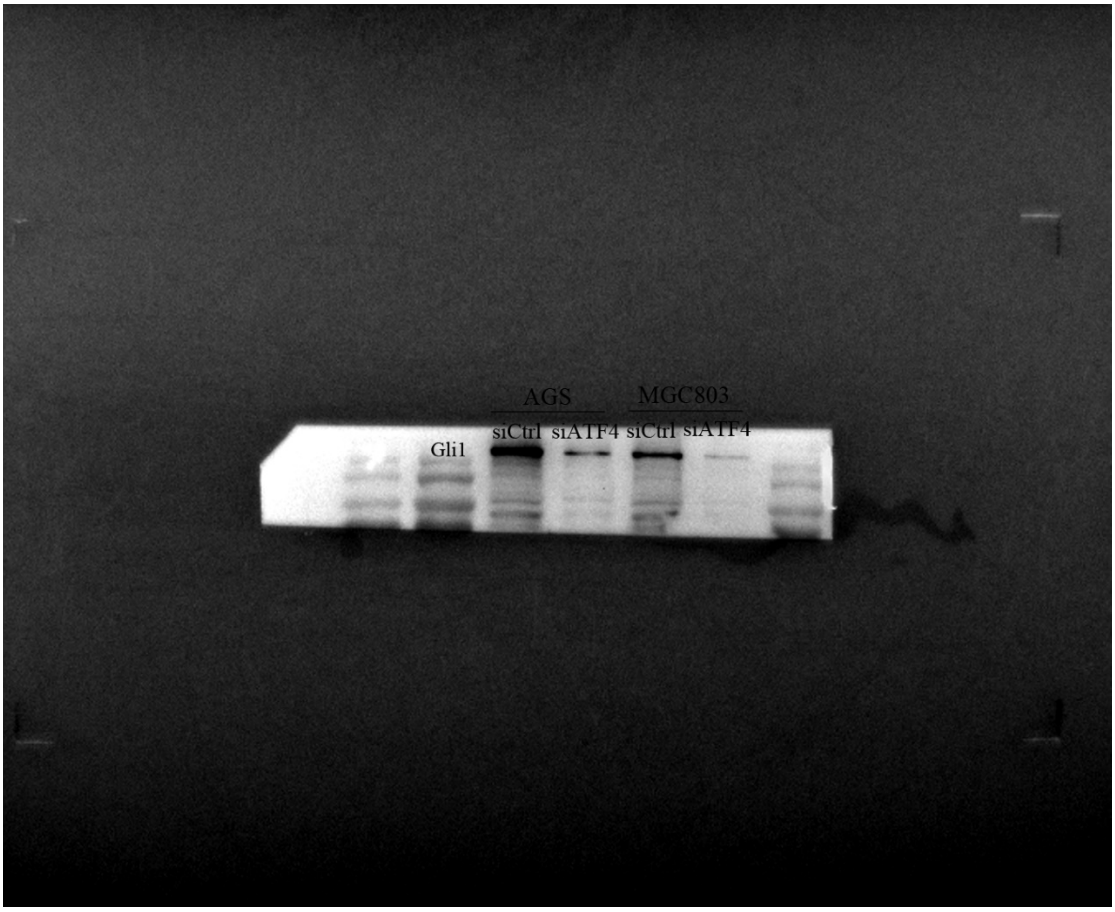
AGS
MGC803
siCtrl siATF4 siCtrl siATF4
Gli1

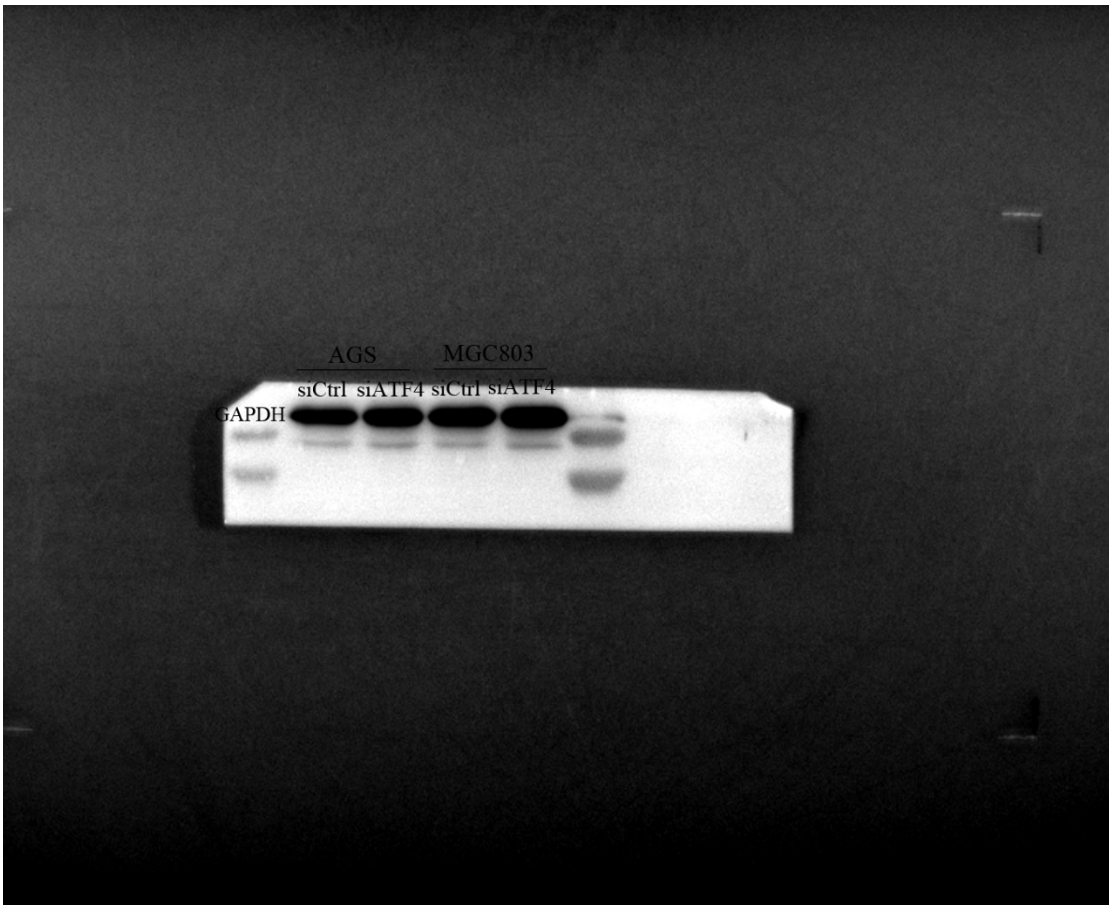
AGS
MGC803
siCtrl
siATF4
siCtrl
siATF4
GAPDH

Figure 4D

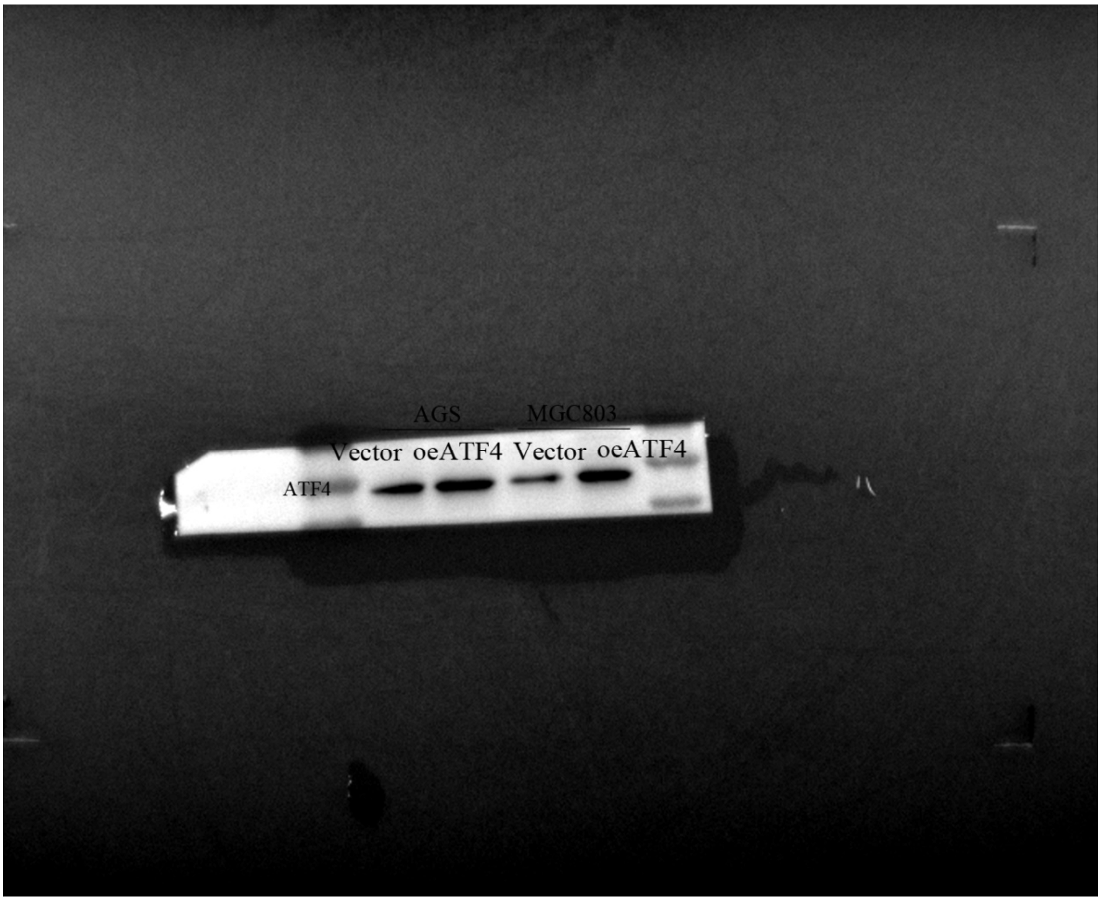

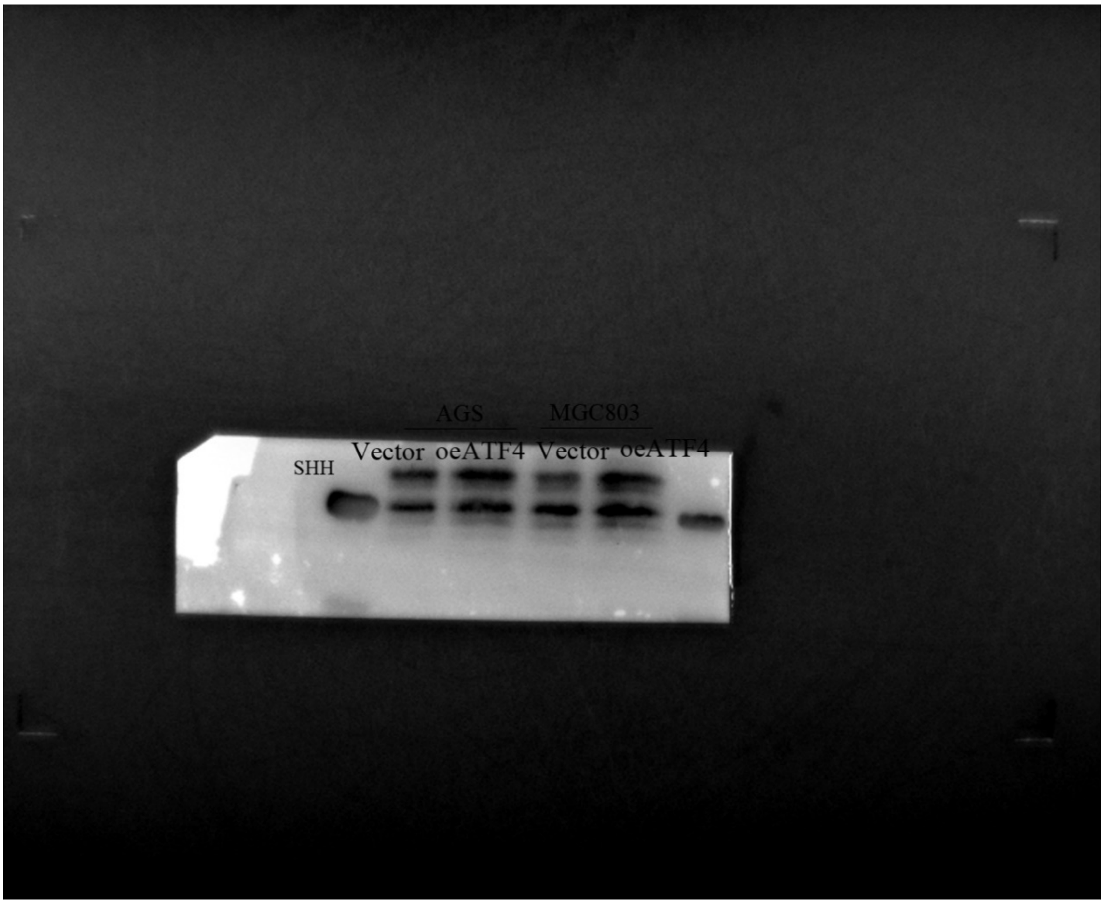
AGS
MGC803
Vector oeATF4 Vector oeATF4
SHH

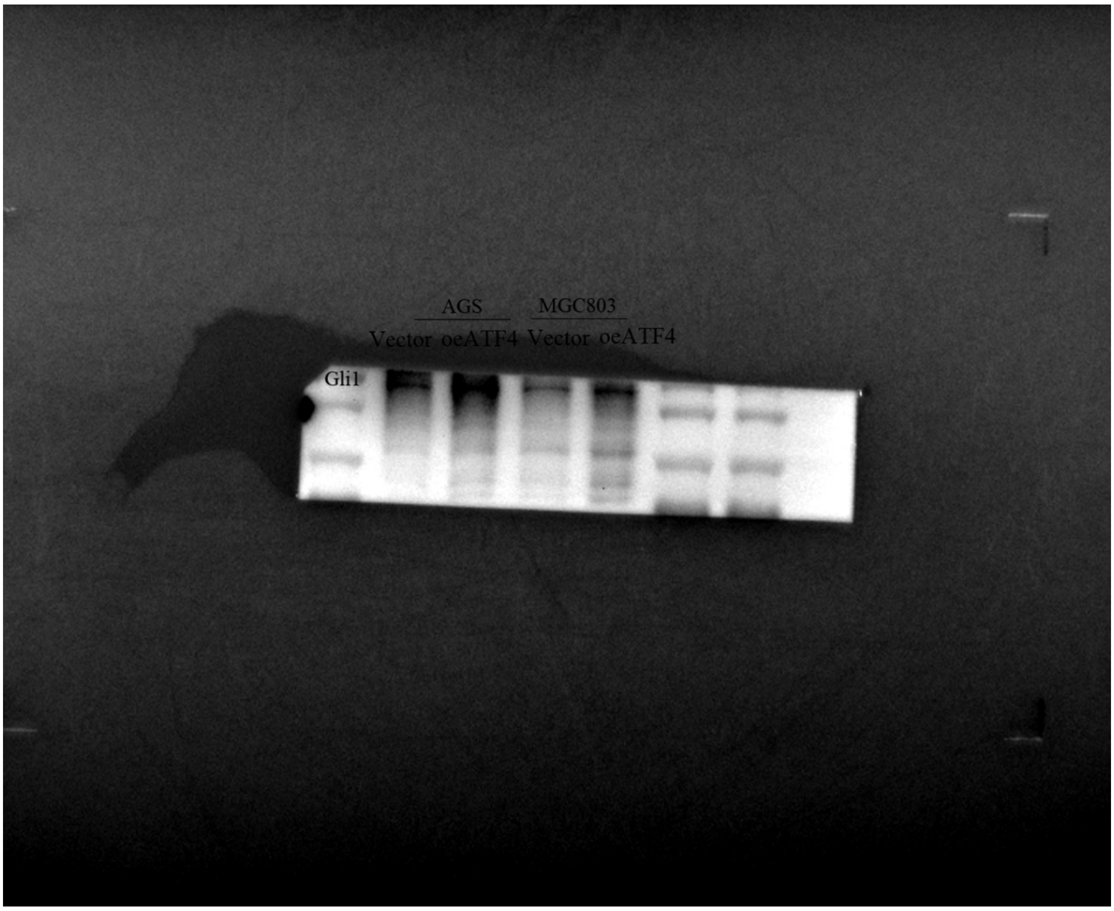
AGS
MGC803
Vector oeATF4 Vector oeATF4
Gli1

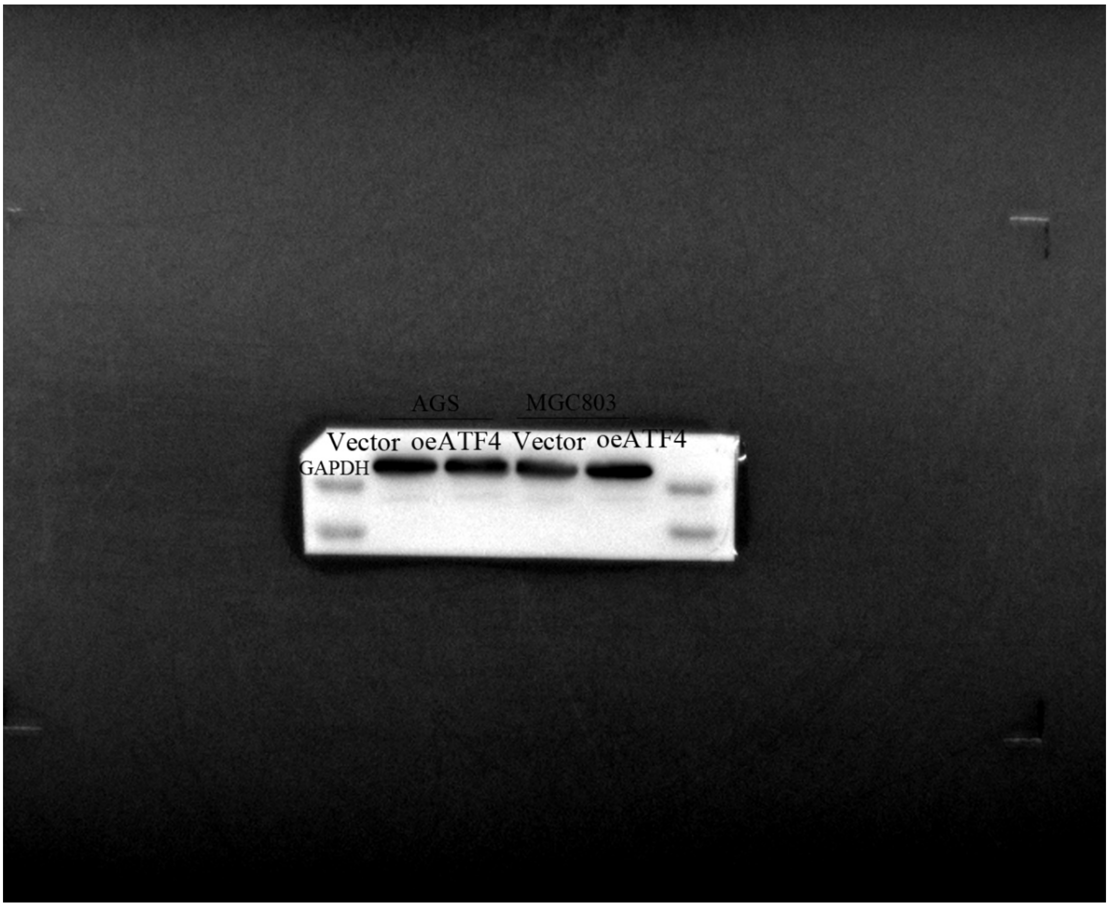
AGS
MGC803
Vector
oeATF4
Vector
oeATF4
GAPDH
